# Genomic Landscape of Primary Mediastinal B-Cell Lymphoma Cell Lines

**DOI:** 10.1371/journal.pone.0139663

**Published:** 2015-11-23

**Authors:** Haiping Dai, Stefan Ehrentraut, Stefan Nagel, Sonja Eberth, Claudia Pommerenke, Wilhelm G. Dirks, Robert Geffers, Srilaxmi Kalavalapalli, Maren Kaufmann, Corrina Meyer, Silke Faehnrich, Suning Chen, Hans G. Drexler, Roderick A. F. MacLeod

**Affiliations:** 1 Leibniz Institute DSMZ, German Collection of Microorganisms and Cell Cultures, Braunschweig, Germany; 2 Jiangsu Institute of Hematology, the First Affiliated Hospital of Soochow University, Suzhou, Jiangsu Province, China; 3 Department of Genome Analysis, HZI, Helmholtz Centre for Infection Research, Braunschweig, Germany; 4 University of Florida, Gainesville, FL, United States of America; University of North Carolina School of Medicine, UNITED STATES

## Abstract

Primary mediastinal B-Cell lymphoma (PMBL) is a recently defined entity comprising ~2–10% non-Hodgkin lymphomas (NHL). Unlike most NHL subtypes, PMBL lacks recurrent gene rearrangements to serve as biomarkers or betray target genes. While druggable, late chemotherapeutic complications warrant the search for new targets and models. Well characterized tumor cell lines provide unlimited material to serve as preclinical resources for verifiable analyses directed at the discovery of new biomarkers and pathological targets using high throughput microarray technologies. The same cells may then be used to seek intelligent therapies directed at clinically validated targets. Four cell lines have emerged as potential PMBL models: FARAGE, KARPAS-1106P, MEDB-1 and U-2940. Transcriptionally, PMBL cell lines cluster near c(lassical)-HL and B-NHL examples showing they are related but separate entities. Here we document genomic alterations therein, by cytogenetics and high density oligonucleotide/SNP microarrays and parse their impact by integrated global expression profiling. PMBL cell lines were distinguished by moderate chromosome rearrangement levels undercutting cHL, while lacking oncogene translocations seen in B-NHL. In total 61 deletions were shared by two or more cell lines, together with 12 amplifications (≥4x) and 72 homozygous regions. Integrated genomic and transcriptional profiling showed deletions to be the most important class of chromosome rearrangement. Lesions were mapped to several loci associated with PMBL, e.g. 2p15 (REL/COMMD1), 9p24 (JAK2, CD274), 16p13 (SOCS1, LITAF, CIITA); plus new or tenuously associated loci: 2p16 (MSH6), 6q23 (TNFAIP3), 9p22 (CDKN2A/B), 20p12 (PTPN1). Discrete homozygous regions sometimes substituted focal deletions accompanied by gene silencing implying a role for epigenetic or mutational inactivation. Genomic amplifications increasing gene expression or gene-activating rearrangements were respectively rare or absent. Our findings highlight biallelic deletions as a major class of chromosomal lesion in PMBL cell lines, while endorsing the latter as preclinical models for hunting and testing new biomarkers and actionable targets.

## Introduction

Primary mediastinal B-Cell lymphoma arises in the mediastinum from transformed thymic B-cells and comprises 2–10% NHL. According to microarray profiling, PMBL is distinct from both germinal center and activated diffuse large B-cell lymphomas (DLBCL) bearing the closest pathological resemblance to classical Hodgkin lymphoma (cHL) nodular sclerosing subtype and mediastinal grey zone lymphoma.

Although PMBL responds initially to chemotherapy subsequent poor prognostic outcomes warrant the search for new targets and disease models [1, 2]. Like cHL, but unlike most NHL subtypes, PMBL lacks recurrent gene rearrangements to serve as diagnostic or prognostic biomarkers or portals to oncogenic drivers, and hence, potential therapeutic targets. PMBL and cHL show alterations at three loci, 2p16 (~50%), 9p24 (~75%), and 16p13 (~45%) [3–5]. Doubt has been cast on the clinical significance of SOCS1 the mooted target at 16p13 [6], while genomic neighbors of JAK2 the preferred candidate at 9p24, namely CD274/PDL1, PDCD1LG2/PDL2 which serve to fatigue reactive T-cells have emerged as alternative targets [7]. Recently, inactivating mutations of PTPN1 have been reported in both PMBL and cHL [8] compounding the list of targets shared by these entities.Low incidence has impeded ascertainment of oncogenomic changes in PMBL [2]. Should key changes be indeed found these may turn out to be rare or cryptic. By permitting in depth studies well characterized tumor cell lines have helped unravel the pathology of such rare or pathologically intractable cancers [9]. In the light of revised PMBL diagnostic criteria four well characterized PMBL cell lines have recently emerged [10]. The advent of forensic DNA profiling promises to dispel the threat of cross contamination widely perceived as a major hindrance [11].

In the quest for PMBL biomarkers and pathological targets we have assembled a panel of PMBL cell lines and documented genomic alterations therein using high density arrays offering circa 40–80x improvements over earlier studies. Candidacies of gene targets were evaluated by parallel expression array profiling and reference clinical data. Several new or unfamiliar potential oncogenomic targets were thus identified. Concordance with clinical data thus observed strengthens the validity of PMBL cell lines as useful models and resources.

## Materials and Methods

### Cell lines

FARAGE was established before 1992 from the lymph node of a 70-year old female at diagnosis of DLBCL sited parasternally [12]. The close similarity of its DNA methylation profile to primary PMBL cells warrants reassignment to that entity [13]. In 1984 KARPAS-1106P and its phenotypically indistinguishable sibling KARPAS-1106A were respectively established from a pleural effusion (at diagnosis) and ascites (during disease progression) of a 23-year old female with “mediastinal lymphoblastic B-NHL” [14]. MEDB-1 was derived in 1981 from the mediastinal mass of a 27-year old male with PMBL (mediastinal B-cell non Hodgkin lymphoma [B-NHL] stage IIb) during relapse [15]. U-2940 was established in 1990 from an 18-year old female diagnosed with B-NHL with mediastinal features subsequent to treatment for cHL [16], but recently reassigned to PMBL [17, 18]. The particulars and culture of these and remaining cell lines are detailled elsewhere [10, 19].

### DNA profiling

To eliminate the risk of cross contamination STR DNA profiling was carried out using fluorescent PCR in combination with capillary electrophoresis as described previously [20]. Briefly, the PowerPlex VR 1.2 system (Promega, Mannheim, Germany) was set to run two-color DNA profiling allowing the simultaneous single-tube amplification of eight polymorphic STR loci plus amelogenin (gender). Loci were amplified by primers labeled with the Beckman/Coulter dye D3 (green; Sigma-Aldrich, Munich/Germany), while the STR loci D16S539, D7S820, D13S317 and D5S818 were amplified using primers labeled with D2 (black). Data (Table A in S1 File) were analyzed with the CEQ 8000 software (Beckman-Coulter, Krefeld, Germany), which enables an automatic assignment of genotypes and automatic export of resulting numeric allele codes into the reference DNA database of the DSMZ.

### Cytogenetic analysis

Cytogenetic analyses were conducted as described previously [21]. Imaging was performed using a Zeiss Axioplan (Oberkochen/Germany) microscope configured to a Spectral Karyotyping (SKY) system (ASI Ltd, Migdal Haemek/Israel). Briefly, bacterial artificial chromosome (BAC) and fosmid clones were obtained from BACPAC Resources, Children's Hospital, Oakland, CA/USA and DNA labeled by nick translation with d-UTP fluors (Dyomics (Jena/Germany). Cell lines were investigated by fluorescence in situ hybridization (FISH) using BAC/fosmid tilepath clones which flanked or straddled loci consistently rearranged in lymphoid neoplasms including, BCL2, BCL6, BCL11A, CCND1, CCND3, IG-H/K/L, JAK2, LMO2, MYC, PAX5, REL.

### Genomic Array Data

CytoScan High Density Arrays which combine oligonucleotide and SNP probes (Affymetrix, High Wycombe/UK) were used to detect genomic copy number alterations/gains/losses (CNA/G/L), losses of heterozygosity (LOH) and unbalanced chromosome translocation breakpoints at high resolution. DNA was prepared using the Qiagen Gentra Puregene Kit (Hilden/Germany). Labeling, hybridization and washing were performed using the recommended kits and CytoScan HD arrays according to the manufacturers protocols. Quality control criteria were those set by the manufacturer. Data were subsequently analyzed using the Chromosome Analysis Suite software version 2.0.1.2 (Affymetrix). This allows access to the Database of Genomic Variants (DGV) (http://dgv.tcag.ca/dgv/app/home) for immediate identification of polymorphic CNV.

### Expression profiling

Total RNA (500 ng) were labelled with biotin according to the 3´ IVT Express Kit (Affymetrix). Circa 7.5 μg of biotinylated cDNA were fragmented and placed in a cocktail containing four biotinylated hybridization controls (BioB, BioC, BioD, and Cre) as recommended by the manufacturer. Samples were hybridized to Affymetrix GeneChip HG-U133 2.0 Plus for 16 h at 45°C. Washing and staining were performed with the fluidics station 450 according to the recommended FS450 protocol. Image analysis was performed on GCS3000 Scanner and GCOS1.2 Software Suite (Affymetrix). Analyses of microarray data were performed using GeneSpring 11.5.1 (Agilent, Santa Clara/CA/USA). Signal intensities (raw data) were log2 transformed and normalized. Comparison datasets were generously provided by Prof. Andreas Rosenwald (Institute of Pathology, University of Würzburg, Germany) or obtained from the BROAD Institute (www.broadinstitute.org). For creation of heat maps we used the software CLUSTER version 2.11 and TREEVIEW version 1.60 (http://rana.lbl.gov/EisenSoftware.htm). For clustering the algorithm was set to Euclidean distance based on p-values confirmed by multiscale bootstrap resampling [22]. For microRNA profiling the Affymetric GeneChip miRNA 2.0 system was used together with samples drawn from the DSMZ cell bank as comparison data sets. For data analysis Affymetrix cel-files were loaded into R/Bioconductor (3.1.0/2.14) using package oligo. External SET2 data are publicly available on GEO (GSM836163, GSM836164, GSM836165). Before principal component analysis (PCA), data were preprocessed, normalized and 10% of the most variable miRNA taken for calculating principal components.

### qPCR

Reverse transcriptase and qPCR used total RNA extracted using the RNEasy Kit (Qiagen). cDNA was subsequently synthesized from 3 μg RNA by random priming, using Superscript II (Invitrogen, Darmstadt/Germany). qPCR was performed by the 7500 Fast Real-time PCR System (Applied Biosystems, Darmstadt/Germany), using SsoFast EvaGreen Real Mastermix (BIORAD, Hercules, CA/USA) and custom designed oligonucleotides (MWG Eurofins, Martinsried/Germany–for sequences see Table B in S1 File). For normalization of expression levels we used TATA box binding protein (TBP). Quantitative analyses were performed in triplicate and repeated twice. The analysis of relative quantitative expression was performed using the Applied Biosystems software (2^ΔΔCt), followed by statistical analysis and data visualization using the R-based ggplot2 package [23].

## Results

### Authentication

STR profiles of PMBL cell lines are given in Table A in S1 File, all testing unique among circa 3000 known cell line profiles provided by the major cell banks (https://www.dsmz.de/services.html). Positive authentication was subsequently provided by cytogenetics (see below).

### Transcriptional clustering and principal component analysis

Unsupervised clustering of microarray expression data were displayed as a dendrogram (S1A Fig) showing that PMBL and cHL cell lines jointly diverge from DLBCL, reflecting their supposed pathologic relationships (replotted from ref. 10). PCA of global microRNA expression arrays again showed the coherence of the PMBL group and their proximity to DLBCL, while segregated from T-cell lymphoma and myelomonocytic neoplasms (S1B Fig). Thus, PMBL cell lines form a coherent and separate entity neighboring cHL, and then DLBLCL.

### Cytogenetics

Consensus ISCN karyotypes [24] of the four PMBL cell lines recorded at the DSMZ and previously by their originators were as follows:

FARAGE: 46(41–46)<2n>XX; no consistent abnormality detected. The originator karyotype was: idem +11. Loss of a chromosome 11 homolog is probably attributable to clonal divergence.

KARPAS-1106P: 49(45–51)<2n>XXX,+X,i(X)(p10),der(X)dup(X)t(X;13)(q25;q11), del(2)(p16p22), der(3)t(2;3)(p25;p14), +9,i(9)(p10),dup(12)(q11q14.1),del(14)(q12q21), del(15)(q11q15),+17, del(18)(q21q22), dup(18)(q12q21),+20,del(20)(q13.1q13.3)x2,add(21)(p13); and that of the originator: 49,XX,der(X)t(X;13;18)(q28;q12;q21),i(Xp),del(2)(p11.2p13), der(3)t(2;3)(p13;p25), +i(9p), ins(12;?)(q13q13),del(14)(q11.2q13),del(15)(q11q15), der(18)t(X;13;18)(q28;q12.;q21),-20, del(20)(q13q13)x2. Thus, both karyotypes are closely similar.

MEDB-1: 47(41–47)<2n>XY,inv(X)(p21;q12),+1,der(1)t(1;14)(p11;q11),t(2;12)(p24;p12),+9, der(10)t(10;20)(q25)(q11),-14,i(21)(q10). The originator karyotype was: 47,XY,inv(X)(p22q13), +der(1)t(1;14)(q10;q10),+9,-14,-21,i(21q), again closely resembling that observed.

U-2940: 45(43–45)X,-X,del(3)(p14p21),del(6)(q13q15),der(7)t(2;7)(q22;p22), dup(12)(q13q22), der(14)t(X;14)(q12;p11),t(16;16)(p12;p13),del(17)(p13); and that of the originator: 45–46,-X,del(3)(p13p21),del(6)(q14q16),del(7)(p11),+i(7)t(2;7)(q23;q31), dup(12)(q12q21), der(14)del(X)(q21.1q21.3)t(X;14)(q11;p11),t(16;16)(p12;p13.3), again resembling that observed.

**Fig 1:**
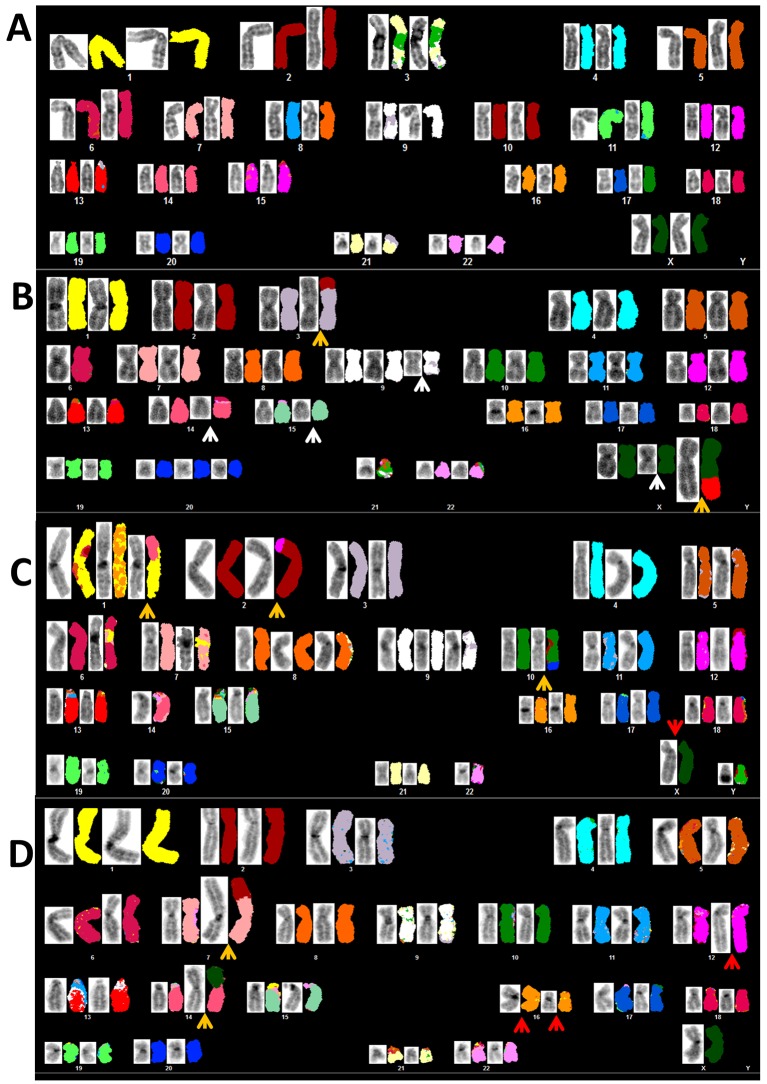
Spectral Karyotyping (SKY): FARAGE (A), KARPAS-1106P (B), MEDB-1 (C) and U-2940 (D). Inverse DAPI G-banding shown left of corresponding pseudocolored SKY. Arrows show deletions (white), duplications (red), and translocations (ochre). After trypsin G-banding (not shown) analyses were extended to prepare consensus karyotypes. Individual metaphases sometimes departed from consensus karyotypes, e.g. note loss of Y-chromosome in MEDB-1. Note absence of key cytogenetic rearrangements and relative lack of rearrangement when compared to cHL cell lines. Thus the salient cytogenetic features of PMBL are essentially negative with respect to neighboring entities, cHL and PMBL.

For representative SKY images see Fig 1A–1D.

Thus, karyotypes of all four cell lines matched the originators’ data, both confirming their stability in vitro and authenticity suggested by their unique STR profiles (Table A in S1 File).

FISH analyses using BAC clones covering lymphoma breakpoints (BCL2, BCL6, BCL11A, CCND1, CCND3, IG-H/K/L, JAK2, LMO2, MYC, PAX5, REL) all tested normal, excluding rearrangements at these loci. Thus, although most PMBL cell line karyotypes bore chromosome translocations, none target known common lymphoma breakpoints.

**Fig 2:**
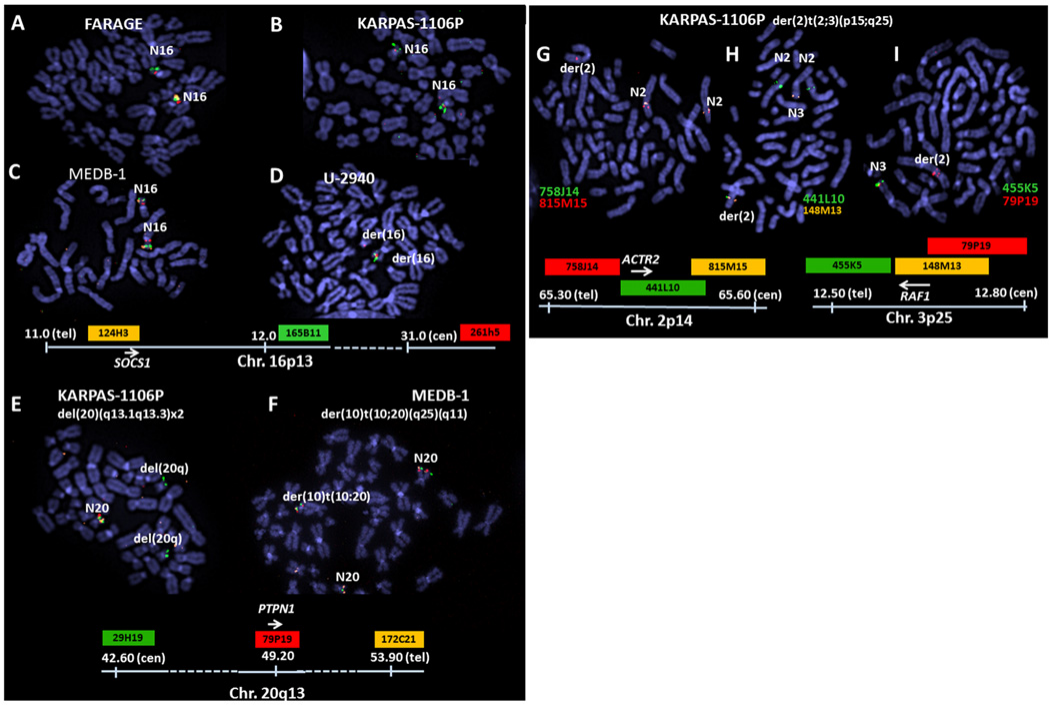
Fluorescence in situ hybridization (FISH) Analysis. A-D: Shows the cytogenetic configuration of focal deletions affecting 16p13 including the SOCS1 locus (arrows) in three PMBL cell lines: monoallelic in FARAGE, biallelic in KARPAS1106P and U-2940 effected by t(16;16) rearrangement, but absent in MEDB-1. E/F: Shows deletions affecting the PTPN1 locus in KARPAS-1106P (E) and MEDB-1 (F). Twin partial chromosome 20 long-arm deletions in KARPAS-1106P effect PTPN1 monosomy, while in MEDB-1 where the gene is mutated this locus escapes deletion despite proximity to a translocation breakpoint therein. G-I: Shows analysis of der(3)t(2;3)(p14;p25) in KARPAS-1106P. The respective breakpoints at 2p14 and 3p25 were placed close to ACTR2 (within clone RP11-441L10) and RAF1 (within RP11-148M13). Coordinates and labelling scheme are shown below. Coordinates (MBp) are from HG19. FISH was performed using tilepath BAC clones.

Cytogenetically visible deletions at 16p13 hosting SOCS1 were present in 3/4 cell lines: monoallelic in FARAGE, biallelic in Karpas-1106P and U-2940, but absent from MEDB-1 (Fig 2A–2D). Only in U-2940 was this rearrangement microscopically visible without FISH. Deletions of 20q13 were present in KARPAS-1106P (Fig 2E) but not in MEDB-1 (Fig 2F). This rearrangement effected PTPN1 monosomy in KARPAS-1106P, while in MEDB-1 where it is mutated [8], PTPN1 escaped deletion.

**Fig 3:**
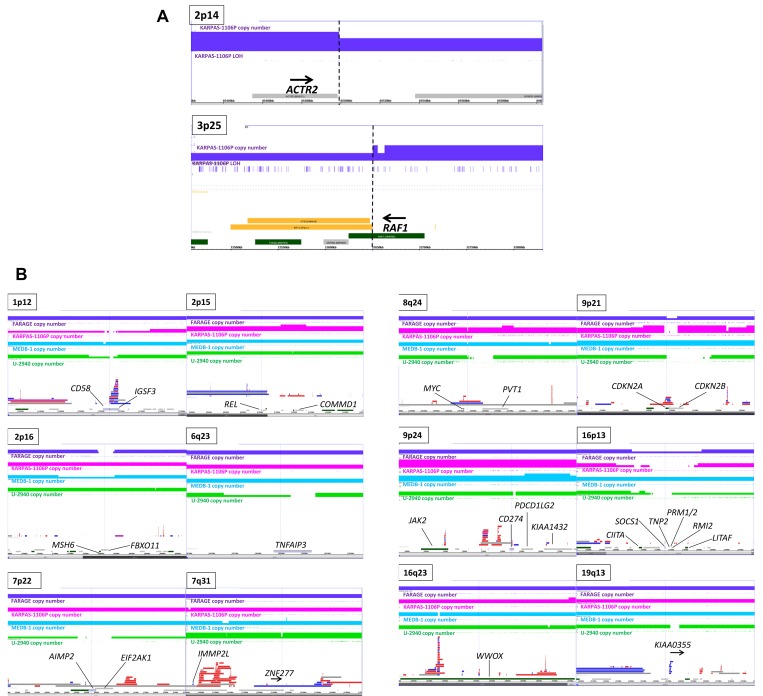
Genomic array data. A: The respective breakpoints and FISH clones at ACTR2 and inside RAF1 of der(2)t(2;3)(p14;p25) in KARPAS-1106P as revealed by genomic arrays expands the FISH data. B: Color coded plots of FARAGE (purple), KARPAS-1106P (pink), MEDB-1 (blue), U-2940 (green)—show genomic copy number (solid) and LOH (barred) at 12 loci (1p12, 2p15, 2p16, 6q23, 7p22, 7q31, 8q24, 9p21, 9p24, 16p13, 15q23, 19q13, together with OMIM genes below. Copy number polymorphic regions (http://dgv.tcag.ca/dgv/app/home) are shown between, listing gains (blue), losses (red), and copy number neutral alterations, such as inversions (gray).

FISH mapped breakpoints of unbalanced der(3)t(2;3)(p14;p25) present in KARPAS-1106P to ACTR2 at 2p25 and RAF1 at 3p14 (Fig 2G–2I). Refinement by genomic array (Fig 3A) placed the 2p14 breakpoint just upstream of the ACTR2 coding region and that at 3p25 inside RAF1 consistent with FISH data. Although ACTR2-RAF1 mRNA fusion has been reported by RNAseq [25], we were unable to detect hybrid mRNA by RT-PCR using published coordinates (S2 Fig). Moreover, formation of the reported chimeric ACTR2-RAF1 mRNA demands an additional cryptic inversion to generate the required ORF.

### Array comparative genomic hybridization (aCGH) and single nucleotide polymorphism (SNP)

Copy number variations/amplifications/deletions (CNV/A/D) and zygosity were investigated with high density combined oligonucleotide SNP arrays. Images depicting each chromosome are shown in S3 Fig Candidate targets of focal CNV, specifically protein coding and RNA genes located within higher level CNA (≥4x) and shared biallelic CND, are respectively listed in Table 1A and 1B, and those of shared monoallelic CND given in Table 1C. Twelve fourfold-or more amplified regions were present (Table 1A) of which four were shared with two or more cell lines, together with a further 12 shared biallelic/monoallelic deletions (Table 1B); these are discussed below. In addition, 49 monoallelic shared deletions were documented (Table 1C).

**Table 1:** Higher-Level Copy Number Alterations and recurrent deletions in PMBL Cell Lines.

Chr. band	Coordinates (KBp)	Cell lines				Genes in region of interest		Co-incident previous studies
		FARAGE	KARPAS-1106P	MEDB-1	U-2940	Protein coding	Noncoding RNA	
**A: Gains (**≥**4x)**								
1p31	72309–72321	2	2	4	2	NEGR1 (part)	-	
1q31	193155–193158	2	2	4	4	B3GALT2 (upstream), CDC73 (part)	-	
2p15	61679–62435	2	4	2	2	XPO1, FAM161A, CCT4, COMMD1, B3GNT2 (part)	-	[6, 26]
2p14	66213–66220	4	3	4	2	SLC1A4 (part)	-	
2q33	207864–208167	2	2	2	5x	KLF7	hsa-mir-2355, hsa-mir-1302-4	[6 9]
3q27	188046–188075	2	2	2	4x	LPP (part), BCL6 (regulatory region)	-	[6, 26]
6q22	125228–126628	2	2	2	4–4.5	NKAIN2 (part), STL, RNF217, TPD52L1, HDDC2, LOC643623,HEY2, NCOA7, TRMT11	LOC643623 MIR5695	
9p24	3087–7795	2	4	3	2	RFX3, GLIS3, PPAPDC2, SLC1A1, CDC37L1, AK3, RCL1, JAK2, INSL4, RLN2, RLN1, CD274, (INSERT), ERMP1, MLANA, IL33, GLDC, KDM4C	hsa-mir-101-2	[6]
9p24 (peak)	5527–5766	3	4	4	2	PDCD1lG2 (part), KIAA1432 (part)	-	
8q24	129061–129153	2	6.5	2	0		PVT1 (part)	[6, 26]
21q11	15639–16173	2	2	4	2	ABCC13,HSPA13, SAMSN1, SAMSN1-AS1, LOC388813	-	
22q12	36679–36694	2	4	4	2	MYH9 (part)	-	
**B: Deletions (bilateral)**								
1p12	117068–117118	2	0	0	2	CD58	hsa-mir-320b	
2p16	48006–48193	0	2	1	2	MSH6, FBXO11	-	
7p22	60267–6095	2	2	2	0	PMS2 (part), AIMP2, EIF2AK1 (part)	-	[26]
8q24	128829–128990	2	3	2	0	PVT1	hsa miR-1204-1208	
9p21	21969–22067	1	0	3	0	CDKN2A, CDKN2B	-	
12q24	133272–133289	2	0	2	2	PXMP2	-	
15q26	100148–100208	2	0	2	2	MEF2A	-	
16p13	10897–1099711169–11682	1	0	2	0	FAM18A (part),CLEC16A (part), SOCS1, TNP2, PRM2, PRM1, RMI2, LITAF	hsa-mir-548h-2	
17q21	42570–42584	1	2	2	0	GPATCH8 (part)	-	
17q21	42596–42599	1	2	2	0	GPATCH8 (5´)	-	
19q13	34737–34809	2	2	3	0	KIAA0355	-	
22q11	183978–18488	2	0	2	2	MICAL3,	hsa-mir-648	
**C: Deletions (unilateral)**								
1p35	25686–25762	2	2	1	1	RHCE	-	[26]
1p13	110479–110484	1	1	2	2	CSF1 (downstream)	-	[26]
1q43	236900–236905	1	2	3	1	ACTN2 (Part; partially overlaps deletion polymorphism)	-	
1q44	241161–241667	1	2	1	2	FH (part)	-	
2p16	50546–50559	2	1	2	1	NRXN1 (part)	-	
2q11	100928–100951	2	2	1	1	LONRF2 (part)	-	
2q12	103099–103104	2	2	1	1	SLC9M (part)	-	
2q12	106384–106390	1	2	3	1	NCK2 (part)17q21	-	
2q14	123282–123303	2	3	1	1	-	-	
2q34	211953–211971	1	1	2	3	-	-	
2q36	223660–223675	2	2	1	1	-	-	
3p21	50627–50638	2	1	2	1	CISH (downstream)	-	[26]
3p14	69532–69554	2	1	1	2	-	-	
3q13	117961–117972	1	1	2	1	-	-	
4p11	48765–48777	2	1	2	1	FRYL (part)	-	
4q13	62870–62877	2	2	1	1	LPHN3 (part)	-	[26]
4q32	-	2	1	1	2	PALLD (non-overlapping losses within same gene)	-	[26]
5q34	167062–167071	2	1	1	2	TENM2 (part)	-	
6q25	149377–149382	2	1	1	2	UST (part)	-	[26]
6q26	164446–164467	1	1	2	2	-	-	
7p21	11162–11167	2	2	1	1	PHF14 (part)	-	[26]
7p15	21579–21584	2	2	1	1	DNAH11 (part)	-	
7p15	27224–27236	1	1	2	2	HOXA11 (part), HOXA11-AS	-	
7p14	30000–30003	2	2	1	1	SCRN1 (part)	-	
7p14	42267–42268	1	2	1	1	GLI3 (part)	-	
7q21	95043–95053	1	2	2	1	PON2	-	
8q22	95398–95399	1	2	1	2	RAD54B(part)	-	
8q22	102670–102674	2	1	1	2	GRHL2 (part)	-	
9p13	37997–37999	2	2	1	1	SHB (part)	-	
10q12	49731–49739	1	1	1	1	ARHGAP22 (part)—adjacent to loss DGV48558)	-	
10q12	51818–51850	2	1	1	2	FAM21B (part)	-	
10q22	73532–73538	1	1	1	2	C10orf54 (5´), CDH23 (part)–adjacent to loss DGV29865	-	
10q22	108236–108253	2	1	1	2	-	-	
11q22	102212–102214	2	2	1	1	BIRC3 (3´), BIRC2 (5´)	-	
11q23	118140–118154	1	2	1	2	MPZL2 (5´)	-	
11q23	-	2	2	1	1	SORL1—non-overlapping losses within same gene	-	
12q23	101601–101611	1	2	1	2	SLC5A8 (part)	-	
13q31	93488–93497	2	3	1	1	GPC5 (part)—adjacent to loss DGV86974	-	
14q22	52271–52276	2	1	2	1	GNG2 (5´)–adjacent to loss DGV87223	-	
14q23	68248–68251	1	1	2	2	ZFYVE26 (part)	-	
14q24	76430–76438	1	1	2	2	TGFB3 (part)	-	
14q31	80199–80210	2	2	1	1	NRXN3 (part)	-	
15q26	101032–101037	1	1	2	2	CERS3 (part)	-	
16p13	7553–7561	1	2	2	1	RBFOX1 (part)	-	
16p13	-	1	2	1	2	LOC283856 (part)—non-overlapping losses within same gene	-	
18q21	5840–358444	1	1	2	2	Gene desert: nearest gene CDH20	-	
19p13	6483–6496	1	1	2	2	TUBB4A (part)	-	
19p11	23557–23564	2	1	2	1	CST9L (5´)	-	
19q13	56076–56085	2	1	1	2	CTCFL (part)	-	

Table lists coordinates (HG19) and hosted loci in PMBL cell lines bearing (A) significant CNV, both gains (≥4x) and (B) losses (null) and both protein coding and noncoding RNA genes located within. Part C lists shared unilateral deletions including non-overlapping deletions in the same genes. Where multiple cell lines are involved coordinates of common affected regions are shown. DGV polymorphisms are excluded.

* CNV approximating those in previous BAC-array studies [6, 26] are denoted accordingly.

**Fig 4:**
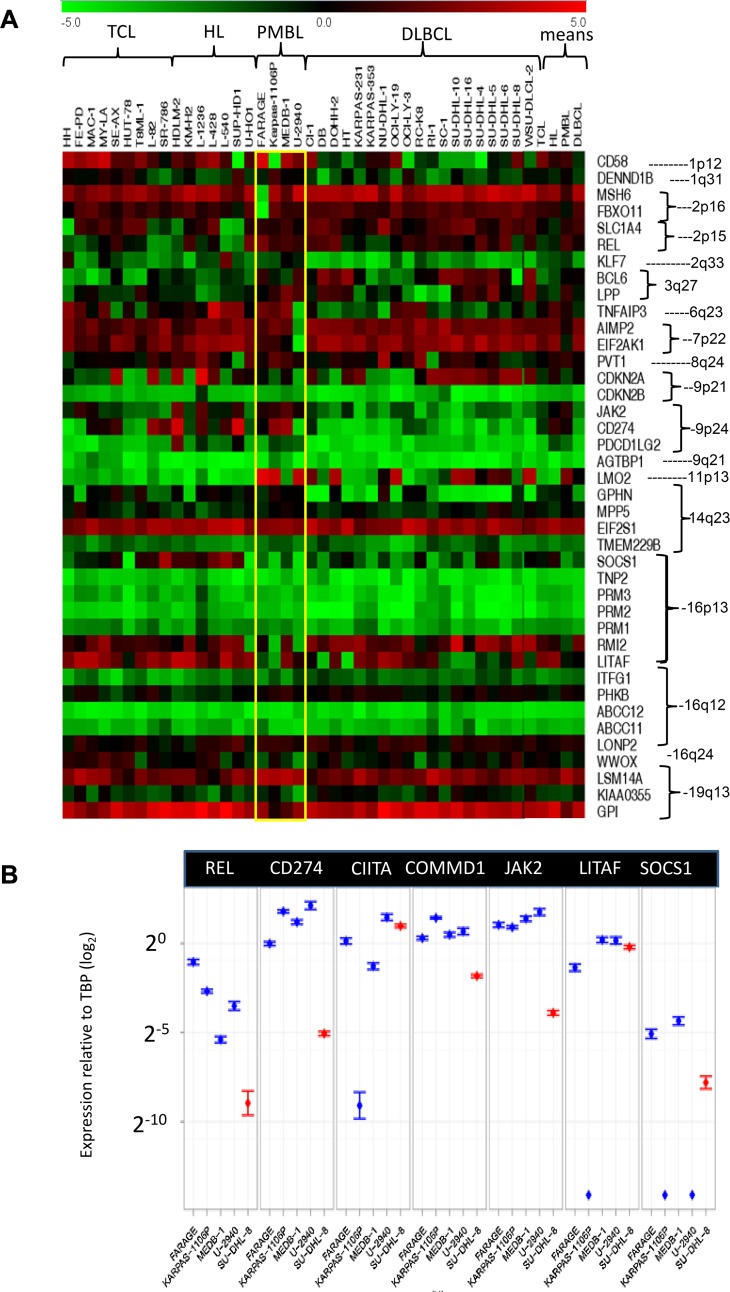
Gene expression at genomically rearranged loci. A: Microarray expression data for genes implicated at CNV (mainly deletions). Note, for example gene silencing accompanying focal biallelic deletions at multiple loci: including, CD58 at 1p12 in KARPAS-1106P; MSH6 and FBXO11 at 2p26 in FARAGE; TNFAIP3, AIMP2 at 6q23 in U2940; EIF2AK1 at 7p22 in U-2940; CDKN2A at 9p21 in KARPAS-1106P and U-2940; CD274 at 9p24 in U-2940; SOCS1 at 16p13 in KARPAS-1106P and U-2940; and KIAA0355 at 19q13 in U-2940. B: Shows qPCR expression of select target genes at recurrent PMBL amplicons (2p15, 9p24) and a deletion (16p13) set against TBP reference in cell lines FARAGE, KARPAS-1106P, MEDB-1, and U-2940 (PMBL) shown blue, alongside reference SU-DHL-8 (DLBCL) shown red. Diamonds indicate undetectable expression. Quantitative data were verified by twofold or more biological replication.

Table 2 lists 72 LOH shared overlapping regions present in two or more PMBL cell lines and genes encoded within. Detailed genomic plots of 12 conspicuously altered loci at 1p12, 2p15, 2p16, 6q23, 6q27, 7p22, 7q31, 8q24, 9p21, 9p24, 16p13, 16q23 and 19q13 are presented in Fig 3B. A corresponding microarray gene expression heatmap covering candidate genes mainly within genomically altered loci is shown (Fig 4A) with select validation by qPCR (Fig 4B).

**Table 2:** Genomic Regions with Shared LOH.

Chr. band	Coordinates (KBp)	PMBL cell lines				Genes in region of interest	
		FARAGE	KARPAS-1106P	MEDB-1	U-2940	Protein coding	RNA/miR
1p36	16321–18176	+	+				-
1p34	31583–31823	-	+	+	+	SDC3, PUM1, NKAIN1, SNRP40,	-
1p34	32266–32755	+	+	+	+	SPOCD1 (part), PTP4A2, KDSRBH1, TMEM39B, KPNA6	-
1p32	51871–52884	-	+	+	+	EPS15, OSBPL9, NRD1, RAB3B, TXNDC12, BTF3L4,ZFY3E9, CC2D1B, ORC1,	MIR761
1p32	52884–53444	-	+	+	-	ZCCHC11	-
1p22	92031–93395	+	+	-	-	HSP90B3P, TGFBR3, BRDT, EPHX4, BTBD8, KIAA1107, C1orf146, GLMN, RPAPq2, GFI1, EVI5, RPL5, FAM69A,	U21, U66
1p21	103783–104829	+	+	+	-	RNPGC3, ACTG1P4, AMY2B, AMY2A, AMY1A,	-
1q44	248127–249177	+	+	+	+	ORL2L13,ORL2M-cluster, ORL2T-cluster,SH3BP5L, ZNF672, PGBD2	hsa-mir-3124
2p16	47460–48198	+	-	+	-	EPCAM, MSH2, KCKN12,MSH6, FBXO11	hsa-mir-559
2p15	61675–62428	-	+	-	-	XPO1, FAM161A, CCT4, COMMD1B3GN2	-
2p14	65230–65231	+	+	+	-	SLC1A4	-
2p11	83872–84993	+	+	+	-	SUCLG1, DNAH6,	-
2q32	195244–196180	+	+	+	+	-	-
3p21	50664–52208	+	+	+	+	MAPKAPK3, DOCK3, VPRBP, RAD54L2,GRM2, IQCF-cluster, RRP9, PARP3, PCBP4, RRP9, ABHD14B, DUSP7, POC1A, ALAS1,	hsa-let-7G, hsa-mir-1
3p11	89163–90486	+	-	+	-	EPHA3	-
4p15	33305–33646	+	-	+	+	-	-
4q31	151969–152275	+	-	+	+	DCLK2, LRBA, RPS3A, SH3D19	U73b
5q12	61391–62553	-	-	+	+	KIF2A, IPO11	-
5q31	130539–130631	+	-	+	+	LYRM7, CDC42SE2	-
6p22	26860–26944	+	+	-	+	-	GUSBP2, LINC00240, LOC100270746
6p22	27212–28454	+	+	-	+	PRSS16, POM121L2, VN1R10P, ZNF204P, ZNF391, ZNF184, HIST1H-cluster, OR2B2, OR2B6, ZSCAN12P1, ZSCAN16, ZNF192, TOB2P1, ZNF193, ZSCAN4, NKAPL, ZNF187, PGBD1, ZNF323, ZSCAN3, ZSCAN12, ZSCAN23	LOC100507173
6q12	64251–64611	+	+	+	-	PTP4A1, PHF3, EYS	-
6q22	125776–126616	-	+	+	+	HEY2,NCOA7,HINT3, TRMT11, CENPW	LOC643623, MIR5695
6q27	138167–138216	-	+	-	+	TNFAIP3	LOC100130476
7p11	56771–57261	+	-	+	+	LOC100130849, ZNF479, GUSBP10,	MIR4283-1, MIR3147
7q36	156187–157470	+	+	-	-	RNF32, LMBR1, NOM1, MNX1, UBE3C, DNAJB6, PTPRN2	hsa-mir-153-2LOC285889LINC00244
8p11	42264–43779	+	+	+	-	VDAC3, SLC20A2, CHRNB3, CHRNA6, THAP1, RNF170, HOOK3, FNTA, HGSNAT, POTEA	-
9p24	2011–2025	+	+	+	+	SMARCA2	-
9p24	5466–5765	-	+	+	+	CD274, PDCD1GL2	KIAA1432
9p24	6655–6712	-	+	+	+	GLDC, KDM4C	-
9p23	10481–10532	+	+	+	+	PTPRD	-
9p21	21435–23074	+	+	+	+	IFNA1, MTAP, CDKN2A, CDKN2B, DMRTA1, FLJ35282	MIR31HG
9q22	94424–95917	-	-	+	+	ROR2, SPTLC1, IARS, NOL8, CENPP, OGN,OMD, ASPN, ECM2, IPPK, BICD2,ANKRD19P, ZNF484, FGD3,SUSD3, C9orf89, NINJ1	-
9q22	97056–98129	+	-	+	-	ZNF169, HIATL1, FBP2, FBP1, C9orf3, FANCC,	hsa-mir-23b, hsa-mir-24-1,hsa-let7-1/7dMIR23BLOC100132077
9q33	125867–126692	+	+	+	+	OR1-cluster, PDCL, RC3H2, ZBTB6, ZBTB26, RABGAP1, GPR21,STRBP, CRB2, DENND1A,	MIR600
10q21	69833–69912	-	+	+	+	MYPN	-
10q22	74721–75779	+	+	-	+	P4HA1, NUDT13, ECD, DNAJC9, TTC18, ANXA7,MSS51, PPP3CB, USP54, MYOZ1, SYNPO2L, BMS1P4, SEC24C, KIAA0913, CAMK2G, PLAU, C10orf55, VCL	-
10q24	104198–104246	-	-	+	+	GBF1, FBXL, CUEDC2,	hsa-mir-146b
10q26	124319–124341	+	+	+	-	DMBT1	-
11p11	46232–47689	+	+	+	+	CREB3L1, DGKZ, AMBRA1, ATG13, ZNF408, F2, CKAP5, LRP4, C11orf49, ARFGAP2, PACSIN3, DDB2, NR1H3, MADDMYBPC3, SPI1, SLC39A13, RAPSN, CELF1,PTPMT1,NDUFS3,NUP160	hsa-mir-3160-1/2, HBII-166hsa-mir-3161
11q13,	71561–72385	-	+	+	+	LOC100133315, RNF121, NUMA1, LRTOMT, LAMTOR1, FOLR3, FOLR1, IL18BP, ANAPC15, FOLR2, INPPL1, PHOX2A, CLPB, PDE2A,	hsa-mir-3165, hsa-mir-139
12p13	313216–315336	-	-	+	+	TEAD4	-
13q12	25725–25758	+	+	-	-	FAM123A	
13q12	30015–31233	-	+	+	-	MTUS2, UBL3, KATNAL1, HMGB1, USPL1	LINC00297, LOC440131LINC00426
14q13	35870–37786	-	+	-	+	NFKBIA, RALGAPA1, BRMS1L, PTSC3, MBIP, SFTA3, NKX2-1, PAX9, SLC25A21, MIPOL1	-
14q23	66556–67954	-	+	+	+	GPHN, MPP5, EIF2S1, TMEM229B	-
14q23	68248–68251	+	+	-	+	ZFYVE26	-
15q13	28297–28560	-	+	+	+	OCAC2, HERC2	-
15q15	41781–41868	-	+	+	+	LTK, ITPKA,RPAP1, TYRO3	-
15q15-21	43928–45972	+	+	-	+	CKMT1A, PDIA3, ELL3, SERF2, SERINC4, WDR76, PIN4P1, FRMD5, CASC4, CTDSPL2, LOC645212,MFAP1, C15orf63, EIF3J, SPG11, PATL2, B2M, TRIM69	hsa-mir-1282
16p13	10901–10971	+	+	-	+	FAM18A,CIITA	-
16p13	11129–11826	+	+	-	+	CLEC16A, SOCS1, TNP2, PRM2, PRM1, RMI2, KITAF, SNN, TXNDC11	hsa-mir-548h-2
16p11	34198–35221	+	-	+	+	UBE2MP1, RN5S411,	hsa-mir-1826RNU6-76, LOC283914LOC146481, LOC100130700, FLJ26245
16q12	47308–48308	+	-	+	+	ITFG1,PHKB, ABCC12, ABCC11, LONP2	MIR648AE2,
16q22	66448–68214	+	-	+	+	BEAN1, TK2, CKLF, CMTM1, CMTM2, CMTM3, CMTM4, DYNC1LI2, CCDC79, NAE1, PDP2, CDH16, FAM96B, CES3, CES4A, CBFB, C16orf70, HSF4, NOL3EXOC3L1, E2F4, LRRC29, SLC9A5, PLEKHG4, LRRC36, TPPP3, HSD11B2, ATP6VOD1, FAM65A, CTCF, RLTPR, ACD, GFPD2, RANBP10, THAP11, NUTF2, EDC4, PSKH1, PSMB10, CTRL, SLC12A4, DPEP3, DDX28, DUS2L, NFATC3	hsa-mir-328
17q21	40514–41964	-	+	+	+	STAT3, PTRF, ATP6VOA1, NAGLU, HSD17B1, COASY, PSMC3IP, FAM134C, MLX, TUBG1, TUBG2, PLEKHH3, CNTNAP1, EZH1, RAMP2, VPS25, CNTD1,BECN1, pSME3, AOC3, AOC4, G6PC, AARSD1, RPL27, RUNDC1, IFI35, RND2, BRCA1, NBR1, DHX8, ETV4, MEOX1, SOST, DUSP3, MPP3, CD300LG, MPP2	LOC100190938,TMEM106A-AS1, LOC100130581, ARL4D, MIR2117,
17q21	43704–45703	+	+	-	+	CRHR1, SPPL2C, MAPT, STH, KANSL1, ARL17A, LRRC37A, NSFP1, LRRC37A2, NSF, WNT3, WNT9B, GOSR2, RPRML, CDC27, MYL4, ITGB3, C17orf57, MRPL45P2, NPEPPS	MGC57346, MAPT-AS1, MIR5089,
17q22	46608–48139	+	+	-	+	HOXB1,HOXB2, HOXB3, HOXB4, HOXB5, HOXB6, HOXB7, HOXB8, HOXB9, PRAC, HOXB13, TTLL6, ATP5G1, UBE2Z, SNF8, GIP, IGF2BP1, B4GALNT2, GNGT2, ABI3, FLJ40194, ZNF652,PHB, NGFR, NXPH3, SPOP, SLC35B1, FAM117A, KAT7, TAC4, DLX4, DLX3, ITGA3,	MIR10A, HOXB-AS3, MIR196A1, MIR3185, LOC294080,
17q23	57929–59172	+	+	-	+	RPS6KB1, TUBD1, HEATR6, CA4, USP32, C17orf64, APPBP2, PPMID, BCAS3	TBC1D3P1-DHX40P1, LOC653653,
19q13	42006–42422	-	+	+	+	CEACAM21, CEACAM4, CEACAM7, CEACAM5, CEACAM6, CEACAM3, DMRTC2, LYPD4, RPS19, CD79A, ARHGEF1	LOC100505495
20q13	46104–58874	-	+	-	+	NCOA3, PREX1, ARFGEF2,STAU1, CSE1L, KCNB1, B4GALT5, PTGIS, SLC9A8, RNF114, SNAI1, SPATA2, UBEV1, CEBPB, LOC284751, PTPN1, PARD6B, BCAS4, ADNP, DPM1, KCNG1, NFATC2, ATP9A, SALL4, ZPF64, TSHZ2, ZNF217, SUMP1P1, BCAS1, CYP24A1, PFDN4, DOK5, CBLN4, MC3R, AURKA, TFAP2C, BMP7, SPO11, RAE1, RBM38, CTCFL, PCK1, ZBP1, PMEPA1, C20orf85, PPP4R1L, RAB22A, VAPB, LOC149773, STX16, GNAS, TH1L, CTSZ, TUBB1, EDN3, PHACTR3, PP1R3D, CDH26, C20orf197, LOC284757	LINC00494, ZNFX1-AS1, U106,HBII-99B, hsa-mir-645, hsa-mir-1302-5, LOC100887755, hsa-mir-3194, hsa-mir-4325, MIR4532, STX16-NPEPL1, hsa-mir-296,
22q13	41303–42236	+	+	+	+	XPNPEP3, RBX1, EP300, L3MBTL2, CHADL, RANGAP1, ZC3H7B, TEF, TOB2, ACO2, POLR3H, PMM1, CSDC2, DESI1, XRCC6, NHP2L1, MEI1, SREBF2,	hsa-mir-1281, hsa-mir-33a,
22q13	50294–50297	+	+	+	-	ALG12	-
Xp22	19115–20973	-	+	+	+	PDHA!, MAP3K15, SH3KBP1, Cxorf23, MAP7D2, EIF1AX, RPS6KA3,	-
Xp11	36152–37511	+	+	+	+	Cxorf59, Cxorf30, FAM47C, PRRG1, LANCL3	-
Xp11	42327–43823	+	+	+	+	MAOA, MAOB, NDP	-
Xq13	46298–46607	+	+	+	+	ZNF673, ZNF674, CHST7, SLC9AS7, RP2, PHF16, RGN, UBA1, INE1, RBM10, CDK16, USP11, ZNF157, ZNF41, ARAF, TIMP1, CFP, ELK1, SYN1, CXXC1P1^1^	SNORA11C
Xq13	74360–75444	+	+	+	+	ABCB7UPRT, ZDHHC15, TTC3P1, MAGEE2	-
Xq22	98574–99573	+	+	+	+	PCDH19, TNMD, TSPAN6	LOC442459
Xq23	109983–111118	+	+	+	+	CHRDL1, PAK3, CAPN6, DCX^1^, DKFZp686DO853, ALG13, TRPC5, TRPC5OS	
Xq26	128870–132178	+	+	+	+	XPNPEP2, SASH3, ZDHHC9, UTP14A, BCORL1, ELF4, AIPM1, RAB33A, ZNF280C, SLC25A14, GPR119, RBMX2, FAM45B, ENOX2, ARHGAP36, IGSF1, OR13H1, MST4, FRMD7, RAP2C, MBNL3,	LOC286467
Xq28	147885–148078	+	+	+	+	AFF2	-

Lists recurrent loci in PMBL cell lines bearing LOH and/or mild coincident CNV with both protein coding and RNA genes located within.

Classical CNA which promotes oncogene activity by increasing mRNA levels occurred only sporadically in PMBL cell lines: singular amplifications, moderate in level (3.5–6.5 fold), were detected in three cell lines (Table 1A). Of 12 CNA seven were focal housing at most one or two genes but were restrained in level and frequency when compared to cHL and DLBCL cell lines. Focal CNA were present in KARPAS-1106P (8q24, Xq11), MEDB-1 (1q31, 9p21) and U-2940 (2q33, 3q28) together with a slightly larger CNA region (ca. 1.2 Mbp) at 6q22. Correlating genomic with microarray expression data (Fig 4A) showed that of four protein coding genes mapping within focal CNA loci, one showed above average (KLF7/2q3), and three near average expression (AGTBP1/9q21, DENN1B/1q31, LPP/3q27), discounting a major role for CNA in gene activation in PMBL cells.

Focal deletions abolish gene expression whether acting alone or in concert with inactivating mutations or epigenetic modifications of residual alleles. Focal null deletions were observed in three cell lines: namely, FARAGE (2p16), KARPAS-1106P (1p12, 1p31, 1p36, 9p21, 16p13, 20q11; plus subclonally at 12q26); and U-2940 (7p22, 8q24, 9p21, 16p11, 19q13) together with 1p12. Of these, three loci bore overlapping null deletions, at 1p12, 9p21 and 16p13. Validation of deletions covering the SOCS1 locus was obtained by FISH for three cell lines, FARAGE, KARPAS-1106P and MEDB-1 (Fig 2A–2C), and for the PTPN1 locus for KARPAS-1106P and MED-B1 (Fig 2E and 2F).

Parallel microarray expression data (Fig 4A) were also used to assess the candidacies of CNV as potential targets and selectively validated by qPCR (Fig 4B and 4C). Independent PMBL patient expression data compared to cHL were extracted from online expression data GSE40160 (extracted from ref. 27) and shown in S4 Fig This exercise highlighted CNV correlated with gene expression, mostly deletions accompanied by gene silencing. These included:

1p12: The CD58 locus was targeted by microdeletion, biallelic in KARPAS-1106P, monoallelic in MEDB-1 and U-2940 (Fig 3B). Expression levels reflected CNV status (Fig 4A). CD58 is also conspicuously silent in PMBL patients (S4A Fig), while neighboring IGSF3 expression remained inconspicuous reflecting that in cell lines (not shown). Although this region carries a polymorphic CNV (Fig 3B), only IGSF3 was affected.

1q31: The DENN/MADD Domain I (DENND1B) gene which is upregulated in PMBL cell lines is amplified in MEDB-1 only (not shown).

2p15: Although genomic array data confirmed the reported CNA in KARPAS-1106P at 2p15, its peak plateau (4n) excluded REL, the mooted target in PMBL (Fig 3B). REL expression, although consistently high in PMBL cells–exceeding that in both cHL and DLBCL–was thus but weakly correlated with CNV. The sole gene inside the amplicon peak region which was consistently upregulated was COMMD1 (Fig 4B), also the most conspicuously upregulated in patients S4A Fig).

2p16: Both MSH6 and the adjacent FBXO11 were deleted in FARAGE (biallelic) and MEDB-1 (monoallelic) (Fig 3) accompanied by dose-related transcriptional downregulation, markedly so in FARAGE (Fig 4A). Both genes are conspicuously silent in PMBL patients and may be deemed candidate tumor suppressor genes (S4A Fig).

3q27: Expression of BCL6 and LPP like DLBCL surpassed that in cHL cells placing PMBL closer to the former entity (Fig 4A). BCL6 copy numbers remained diploid (not shown) although expression was raised in FARAGE and U-2940 and lowered in KARPAS-1106P and MEDB-1. Interestingly, BCL6 was conspicuously well expressed in PMBL patients (S4A Fig).

6q23: Biallelic deletion affecting TNFAIP3/A20 (Fig 3B) accompanied conspicuous silencing of this gene in U-2940 cells (Fig 4A). Silencing also affected some DLBCL and TCL cell lines (Fig 4A). LOH at this locus was present in KARPAS-1106P (not shown) where expression levels were intermediate (Fig 4A). The same cell line reportedly carries an inactivating TNFAIP3 mutation [28]. TNAFAIP3 silencing was also apparent among PMBL patients (S4A Fig).

7p22: Focal biallelic deletion of AIMP2 and EIF2AK1 (Fig 3B) accompanied conspicuous silencing of both genes in U-2940 (Fig 4A). Of these, EIF2AK1 evidenced the greater silencing in PMBL patients (S4A Fig).

7q31: A complex pattern of deletion and amplification was observed. ZNF277 was downregulated in U-2940, and IMMP2L in MEDB-1 (not shown) which respectively bore focal monoallelic deletions at these loci (Fig 3B). In PMBL patients both genes are downregulated in patient subsets (S4A Fig).

8q24: A complex CNV pattern was observed around PVT1 and the associated miR cluster while excluding MYC (Fig 3B). The centromeric microdeletion present in all four cell lines is apparently non-polymorphic. Biallelic deletion in U-2940 and four-fold amplification in KARPAS-1106P affecting PVT1 were reflected transcriptionally (Fig 4A), while the embedded miR cluster (miR-1204-1208) remained inconspicuously silent throughout in miR arrays (not shown). Likewise, PMBL patients expressed widely fluctuating PVT1 levels (S4B Fig).

9p21: In KARPAS-1106P and U-2940 biallelic, and in FARAGE monoallelic, focal deletions affected the adjacent CDKN2A/B loci (Fig 3B). Although trisomic, the locus in MEDB-1 also evidenced LOH (not shown). Gene expression data showed silencing of CDKN2B in all cell lines irrespective of ploidy, and of CDKN2A except in MEDB-1, but conspicuously so in U-2940 and KARPAS-1106P (Fig 4A). qPCR confirmed CDKN2A/B silencing (*not shown*). In patients as in cell lines CDKN2A was conspicuously silenced, and CDKN2B unevenly so (S4B Fig). Collectively, these findings raise the spectre of epigenetic controls at this locus, such as silencing by DNA methylation.

9p24: At this site of recurrent CNV ploidy gains were ubiquitous in both PMBL and cHL, sparing only FARAGE. Tetraploidy in KARPAS-1106P was extended plateau-like, but focal in MEDB-1 cresting a wide triploid region, while a microdeletion in U-2940 impinged CD274. Consensus gains included PDCG1LG2 and KIA1432 (Fig 3B). There was no clear correlation between CNA and transcriptional upregulation, as both JAK2 and CD274 which showed unambiguous upregulation (Fig 4A and 4B) shunned the amplicon peak. Thus, while endorsing the existence of a 9p24 amplicon in PMBL its relationship to JAK2 remains enigmatic, the combined CNA and expression data marginally favoring CD274 while failing to exclude multiple targeting.

16p13: Genomic deletions, monoallelic in FARAGE, biallelic in KARPAS-1106P/MEDB-1/U-2940, covered 6 transcriptionally silenced genes at 16p13: namely, CIITA, LITAF, SOCS1, RM1/2 and TNP2, (Fig 3B). On expression arrays the flanking candidates CIITA and LITAF undercut cHL, DLBCL or T-ALL cell lines (Fig 4A). According to qPCR data, SOCS1 showed the most consistent silencing, just ahead of LITAF which lay outside the CDR (Fig 4B). Uniquely, in U-2940 a cytogenetic rearrangement t(16;16) accompanied deletion (Fig 1D). In patients, of these candidates only LITAF was conspicuously underexpressed, and RMI2 but moderately so (S4B Fig). CIITA/MHC2TA expression remained nondescript in both settings (Fig 3C and Fig 4B).

16q23: Biallelic and monoallelic deletions affecting the common fragile site FRA16D gene WWOX were present in MEDB-1 and U-2940 (Fig 3B) correlated with modest expression seen therein (Fig 4A). Interestingly, both deletions are intronic and perfectly correspond to known polymorphisms (Fig 4A). In PMBL patients WWOX is conspicuously downregulated (S4C Fig).

17p13: Although TP53 may be inactivated in PMBL, this locus displayed neither copy number losses nor LOH. Expression remained unperturbed (not shown).

17q21: GPATCH8 uniquely bore a singleton biallelic deletion in U-2940 which abolished transcription, while the monoallelic deletion in FARAGE accompanied depressed microarray expression therein (not shown).

19q13: A biallelic focal deletion was present in U-2940 (Fig 3B) accompanied by conspicuous silencing of KIAA0355 (Fig 4A) seen also in patients (S4C Fig).

21q12: A discrete fourfold amplicon of circa 15 Kbp was present in both KARPAS-1106P and MED-B1 (Table 1A) which although apparently identical in both cell lines is not hitherto recorded as a polymorphism. This region includes part of the coding region of MYH9 which was, however, inconspicuously expressed in both patients and cell lines (not shown).

In addition to biallelic deletions which ipso facto enforce gene silencing, 49 shared monoallelic deletions (SMD) were recorded (Table 1C). On average shorter than biallelics, these sometimes shared identical coordinates and may represent unrecorded polymorphisms. To assess their significance SMD were also checked against microarray expression. This exercise revealed two examples correlating with gene expression, one positively (2q12/NCK2) and one negatively at 10q12/FAM21B, i.e. within expected stochastic noise levels.

### Losses of heterozygosity (LOH)

LOH are thought to promote cancer by exposing recessive gene mutations or epigenetic silencing modifications among tumor suppressor genes. Alternately, LOH may activate oncogenes by silencing their cognate micro-RNAs. In total 72 shared LOH were recorded the most frequent and widespread alterations present (Table 2). Most LOH were extensive covering multiple genes, hampering target identification. A minority were focal pinpointing candidate gene targets within a few loci, e.g. at 6q23 (TNFAIP3) and 9p24 (CD274)–both targeted separately by focal deletions in other cell lines (Figs 3B and 4A and 4B). LOH also occurred independently of deletions, e.g. at 16q12 FARAGE, MEDB-1 and U-2940 (Table 2) negatively correlated with gene expression at this locus (Fig 4A): of potentially inactivated genes inside the common LOH region (EIF2S1, GPHN, and MPP5) were also underexpressed in PMBL patients (S4B Fig). In the same three cell lines LOH was also observed at 16q12 where ITFG1 and PHKB were downregulated both in the affected cells (Fig 4A) and in PMBL patients in general (S4 Fig). Singleton LOH was accompanied by conspicuous LMO2 silencing in MEDB-1 covering the 3´ distal regulatory region (Fig 4A). In the PMBL patient series LMO2 was highly expressed throughout highlighting downregulation in MEDB-1 (S4B Fig). LOH at 14q23, in KARPAS-1106P, MED-B1 and U-2940 was seemingly accompanied by downregulation of GPHN and MPP5 (Fig 4B). In PMBL patients, however, the same genes were conspicuously silent as was the adjoining EIF2S1 (S4B Fig). While microRNA loci also fell within genomically altered regions offering a possible explanation for upregulation accompanying LOH—parallel miR expression arrays provided no evidence that these impacted transcription (not shown).

## Discussion

By integrating genomic with parallel transcriptional data sets we both documented genomic alterations in PMBL cell lines and prioritized gene candidates for further assessment. Given the degree to which cell lines portray the emerging oncogenomic picture of PMBL we conclude that they provide suitable resources for pursuing further investigations into this enigmatic neoplasm. Shared impactful changes were shown to feature in two or more cell lines forming patterns of recurrence warranting further study. Although their rearrangement spectrum partially overlaps neighboring entities cHL and DLBCL, PMBL cell lines stand apart bearing far fewer rearrangements than the former, while lacking the recurrent B-cell oncogene translocations of the latter. These evidences plus its snug transcriptional niche close to both neigboring entities show that the PMBL cell lines fill a unique oncogenomic niche consistent with their attribution to PMBL.

The likely import of deletions in PMBL was first bestowed by low density array studies [6, 26]. While broadly consistent with these groundbreaking studies (Table 1), the increased sensitivity afforded by high density arrays allowed detection of an additional layer of CNV, mainly unilateral deletions invisible to BAC arrays. The CNV impacting gene expression the most were, however, bilateral deletions affecting protein coding genes. Top genes/loci verifiably impacted by deletions and/or LOH included MSH6/FBXO11 at 2p16 (in two cell lines), TNFAIP3 at 6q23 (three), CDKN2A at 9p21 (four), and SOCS1 (four) together with LITAF (three) at 16p13—all plausible oncogenic agents, including some already known in PMBL. Although additional CNV were detected, notably the unilateral short CND discussed above, their targets and impact, remain doubtful after assessment with parallel expression data. The significance of other hitherto uncharacterized deletion candidates, e.g. the ubiquitously expressed GPATCH8 at 17q21 and KIAA0355 at 19q13, also awaits further study.

Among validated candidates, SOCS1 deletion/mutation in KARPAS-1106P and MEDB-1 has been already described where it delays JAK2 degradation to allow pSTAT6 stockpiling [29, 30]. Our findings add FARAGE and U-2940 to the list of cell lines bearing 16p13 deletions and confirm concurrent SOCS1 inactivation throughout. Our data also indict neighboring LITAF (and possibly RMI2) as collateral targets. While SOCS1 remains the prime target of 16p13 deletions, LITAF (lipopolysaccharide-induced TNF-alpha factor)—a DNA binding protein which promotes cytokine production including TNF-α, seems to be affected by this lesion. Promoter methylation and biallelic deletion of LITAF has been reported in DLBCL [31]. Here we show that LITAF is both deleted and downregulated in PMBL cell lines while conspicuously silenced in PMBL patients (S4C Fig). LITAF has been identified as a BCL6 target in mature B-cell lymphomas where it regulates autophagy [32]. Together with STAT6, LITAF governs CCL2 expression via NFKB1, and its ancient genomic proximity to SOCS1 may reflect a need for co-regulation. A tumor suppressor role for LITAF has also been reported [33], and this role invites further study in PMBL. Nearby RMI2 is a component of the BLM complex via processing of Holiday junctions formed after DNA repair [34] and its role, if any, in PMBL requires to be established.

In 21% DLBCL cases the immunosurveillance marker CD58 (LFA3) is inactivated [35], as reflected by our cell line (Fig 4) and patient data (S4A Fig) consistent with previous reports [4, 35]. Loss of CD58 expression as seen in KARPAS-1106P recalls an immune escape mechanism reported in DLBCL [36].

FBXO11 at 2p16 forms a complex which targets BCL6 for ubiquitylation and proteasomal degradation in DLBCL [37]. Biallelic deletion and silencing of FBXO11 among lymphoma cell lines occurred uniquely in FARAGE along with raised BCL6 expression. MSH6—co-deleted and silenced along with FBXO11—is subject to inactivation in DLBCL associated with microsatellite instability, increased structural rearrangement and altered mutation signatures [38]. PMBL cell lines also carry mutations in other genes affecting genome stability (Ehrentraut et al., in preparation). BCL6 and FBXO11 are also weakly expressed in PMBL cell lines and merit clinical evaluation.

Centromeric of FBXO11 lies REL at 2p15 which though highly expressed in PMBL cell lines (Fig 4A and 4B) falls outside the CNA region in KARPAS-1106P implying deregulation other than via CNV. Interestingly, genome wide association studies have shown that single polymorphic base changes, at 2p15 near REL and 8q24 near PVT1 where recurrent CNV were detected (Fig 3B), increase the risk of cHL [39]. Alhough COMMD1 activation is less emphatic, it lies within the amplicon peak and is also well expressed in PMBL patients. COMMD1 shortens survival in DLBCL [40], perhaps via deregulation of NFκB [41], and its candidacy merits consideration alongside REL whose overexpression is firmly linked to CNA at 2p15 [42].

TNFAIP3 inactivating mutations occur widely in DLBCL, cHL and in PMBL patients and have been reported in KARPAS-1106P [28, 43]. Mutations inactivating TNFAIP3 are also present in MEDB-1 (Ehrentraut et al., in preparation). These findings, together with the expression data pinpoint TNFAIP3 as a key tumor suppressor gene. Inactivation has been described in a variety of T/B-cell lymphomas and leukemias where it is also thought to promote activation of NFκB [44], a gene well expressed in all 4 PMBL cell lines.

Although deletions affecting 9p21 are widespread in aggressive leukemia and have been described in DLBCL, genes at this locus emerge relatively unperturbed in PMBL [45]. Here lie the coding regions for CDKN2A/B which yield several transcript variants, including p14/ARF from a different reading frame. CDKN2A/B foster self-renewal and, though epigenetically silenced throughout hematopoiesis, subsequently remain poised for reactivation by oncogenic stress. Oncogenomic losses posited to affect this locus are attended by special caveats concerning polymorphism (both gains and losses) and the possibility of immortalization artifact. However, clinical data show that CDKN2A inactivation is widespread in PMBL patients (S4B Fig) buttressing the cell line data.

Our data also show that the posited genomic upregulation of JAK2 which outlies the CNA peak also warrants further study. Although JAK2 is constitutively phosphorylated in MEDB-1 at least [46], “smoking gun” mutations or translocations remain unreported in PMBL and cHL alike [47]. Nevertheless, the hypersensitivity of PMBL cell lines to JAK2 inhibitors has been taken to imply oncogene addiction [48]. JAK-STAT signalling in PMBL is relayed via STAT6, itself constitutively activated in both KARPAS-1106P and MEDB-1 recalling cHL and DLBCL [46, 49]. BCL6 silencing has been identified as a downstream target of JAK-STAT signalling [50] further highlighting this pathway as an actionable target in PMBL. Again, our data highlighted an alternative target at 9p24, CD274/PDL1, which confers immune privilege by binding the PD1 receptor on reactive CD8+ T-cells and thus inhibits proliferation [51], offering a novel candidate for blockade therapy [52].

High density oligonucleotide/SNP arrays remove some bias inherent in BAC arrays while offering improved resolution, parallel LOH data, and—thanks now to the continually updated Database of Genomic Variants—the ability to sift out natural polymorphic CNV. Integrating genomic and transcriptional microarray data allows prioritizing of gene candidates targeted by genomic rearrangements at a first pass level. By delineating ever shorter regions of interest, high density arrays simplify candidate ranking. Finding little evidence that chromosomal translocations impact cancer gene alterations, our findings pinpoint bilateral microdeletions as the predominant class of impactful genomic lesion in PMBL cell lines. Thus our findings strengthen the case for applying high density arrays to clinical samples. And while LOH was in a few cases also correlated with gene silencing, its likely transcriptional—hence oncogenic—consequence remains doubtful. Parallel studies addressing the impact and role of gene mutations in PMBL cell lines are nearing completion in this lab, while those addressing the role of epigenetic modifications, such as DNA methylation, in gene regulation in PMBL are underway.

This study combined high density oligonucleotide/SNP arrays with cytogenetics to profile the genomic features of PMBL cell lines revealing subtle genomic changes therein, notably short genomic deletions. By integrating genomic lesions with global cell line and patient expression data we shortlisted potentially impactful gene targets therein, comprising known, suspected and hitherto inconspicuous candidates. The current investigation endorsed the validity of this panel to serve as an oncogenomic resource and scaffold in pursuit of novel biomarkers and actionable gene targets in PMBL and evaluate “intelligent” therapies directed against specific lesions in cells bearing them.

## Supporting Information

S1 Fig Transcriptional Clustering and Principal Component Analysis (PCA). A: Transcriptional profiling of PMBL and other hematopoietic cell lines was used to construct a cluster diagram–(updated from ref. 10)—of mRNA expression. The AU value (printed red) gives the "approximately unbiased" p-value, which is calculated by multiscale bootstrap resampling. The bootstrap probability value is less stringent than AU value when testing significance. Clusters (edges) with high AU values are strongly correlated. Results are shown as a dendrogram, showing different expression profiles as early dividing branches. B: PCA plot of microRNA expression. Note discrete clustering of both mRNA and miR expression showing that PMBL cell lines occupy a unique niche apart from other hematopoietic entities. Abbreviations: AML, acute myeloid leukemia; DLBCL, diffuse large B-cell lymphoma; erythro-megakaryocytic leukemia; ITL, immortalized T-cell; PMBL, primary mediastinal B-cell lymphoma; TCL, T-cell lymphoma.
(TIF)

S2 Fig RT-PCR to detect ACTR2-RAF1 fusion in KARPAS-1106P. Shows absence of product yielded for ACTR2-RAF1 fusion suggested by genomic breakpoints and reported recently [25]. Amplification of ETV6 served as positive control for confirmation of cDNA quality. NTC: no template control. Control cell line HL-60 is derived from a patient with acute myeloid leukemia.
(TIF)

S3 Fig Whole chromosome copy number plots showing LOH. See legend to Fig 3.
(PDF)

S4 Fig Select gene expression in PMBL patients (GSE40160). Shows global microarray expression for select genes in PMBL patients compared to cHL. Data extracted from ref [27].
(PDF)

S1 File Contains the following: Table A. STR Profiling Data. Table B. Primers for RqPCR. (DOCX)
