# Supplementary figures and images for: Genomic Landscape of Primary Mediastinal B-Cell Lymphoma Cell Lines
Source: PLoS One. 2015 Nov 23;10(11):e0139663. doi: 10.1371/journal.pone.0139663 (PMC4657880; doi:10.1371/journal.pone.0139663)

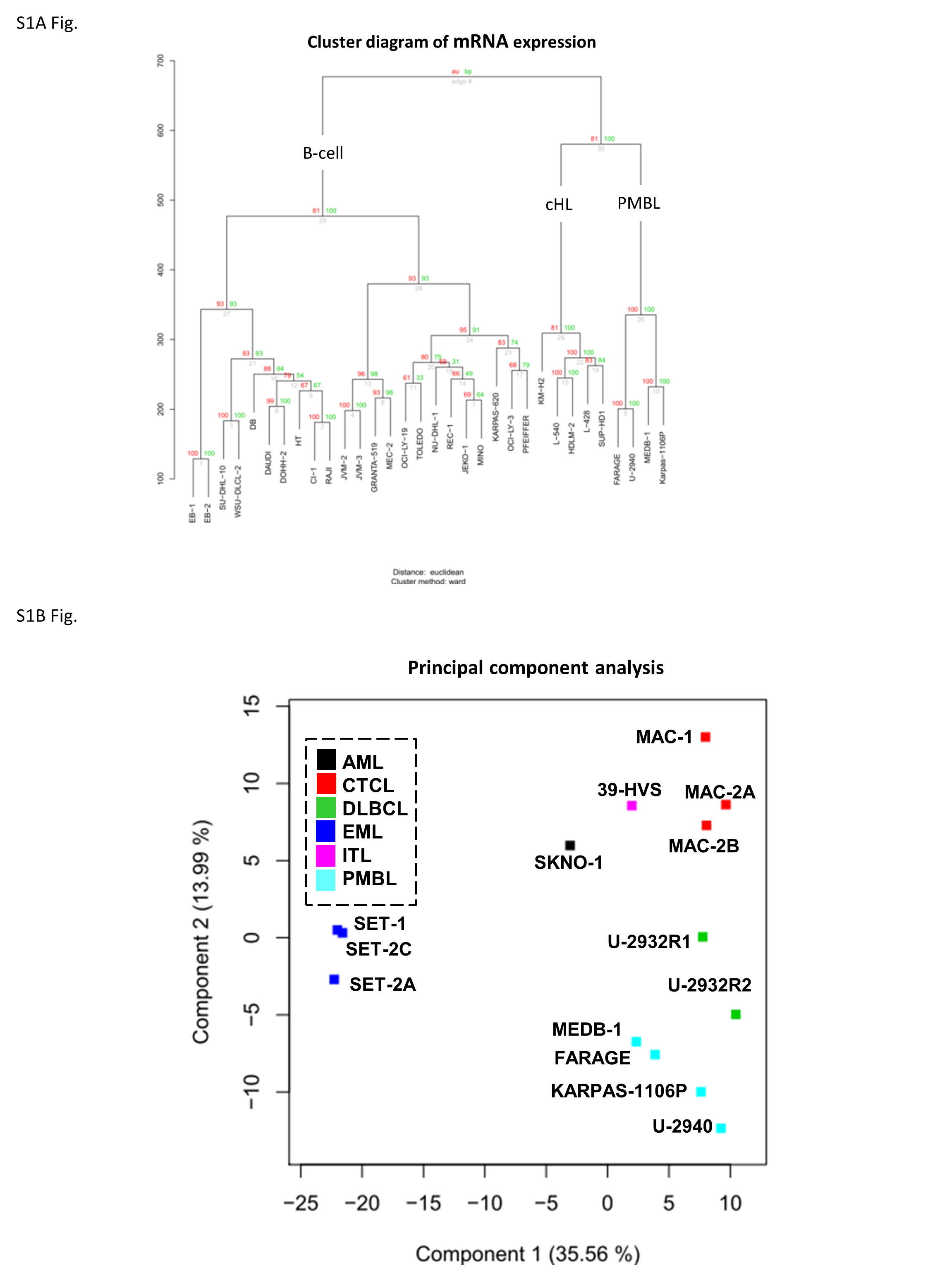

Supplement: S1 Fig — A: Transcriptional profiling of PMBL and other hematopoietic cell lines was used to construct a cluster diagram–(updated from ref. 10)—of mRNA expression. The AU value (printed red) gives the "approximately unbiased" p-value, which is calculated by multiscale bootstrap resampling. The bootstrap probability value is less stringent than AU value when testing significance. Clusters (edges) with high AU values are strongly correlated. Results are shown as a dendrogram, showing different expression profiles as early dividing branches. B: PCA plot of microRNA expression. Note discrete clustering of both mRNA and miR expression showing that PMBL cell lines occupy a unique niche apart from other hematopoietic entities. Abbreviations: AML, acute myeloid leukemia; DLBCL, diffuse large B-cell lymphoma; erythro-megakaryocytic leukemia; ITL, immortalized T-cell; PMBL, primary mediastinal B-cell lymphoma; TCL, T-cell lymphoma. (TIF) [file pone.0139663.s001.tif]

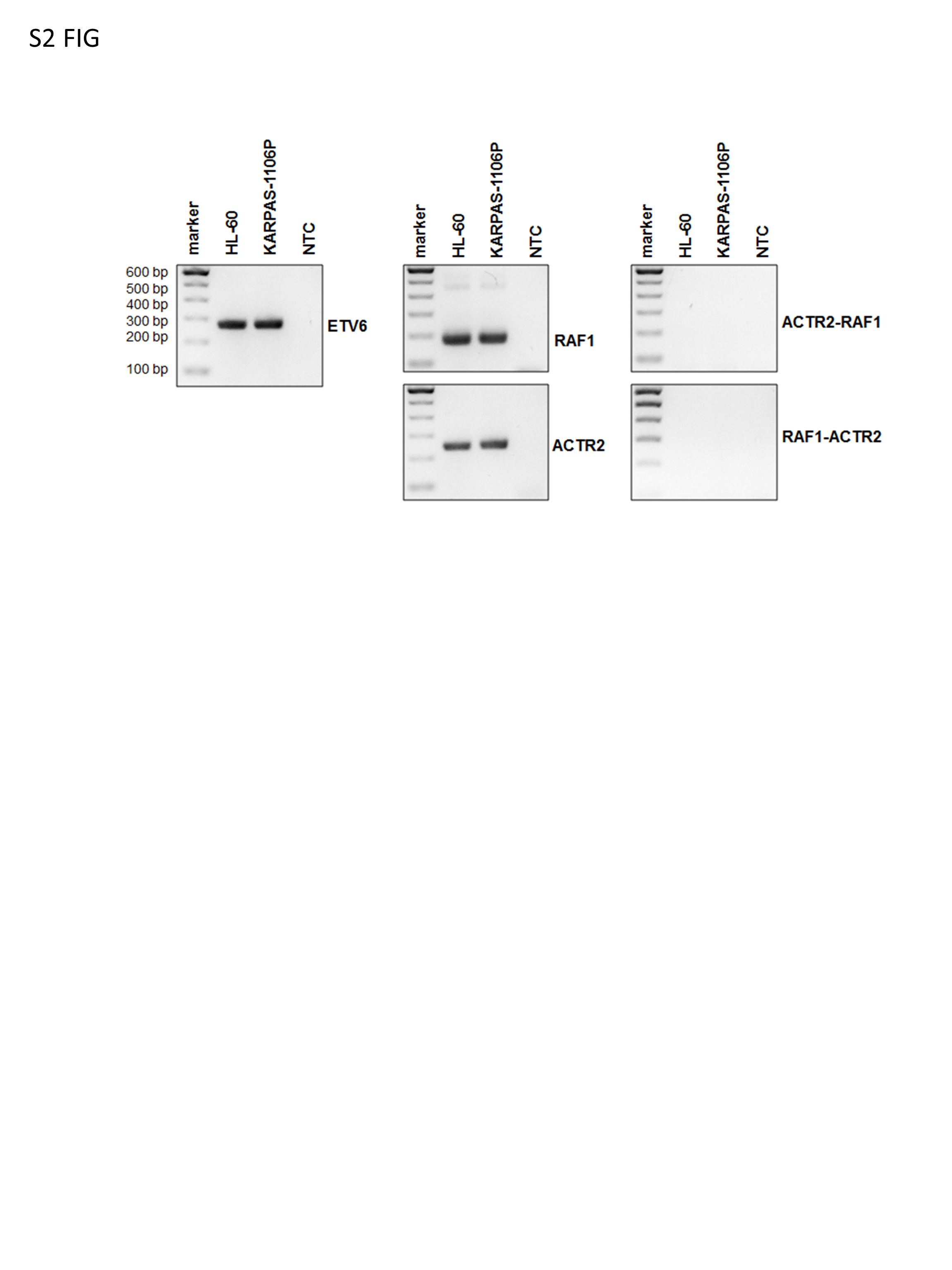

Supplement: S2 Fig — Shows absence of product yielded for ACTR2-RAF1 fusion suggested by genomic breakpoints and reported recently [25]. Amplification of ETV6 served as positive control for confirmation of cDNA quality. NTC: no template control. Control cell line HL-60 is derived from a patient with acute myeloid leukemia. (TIF) [file pone.0139663.s002.tif]
