# Supplementary material for: Genomic Landscape of Primary Mediastinal B-Cell Lymphoma Cell Lines
Source: PLoS One. 2015 Nov 23;10(11):e0139663. doi: 10.1371/journal.pone.0139663 (PMC4657880; doi:10.1371/journal.pone.0139663)

Suppl. Fig. 3

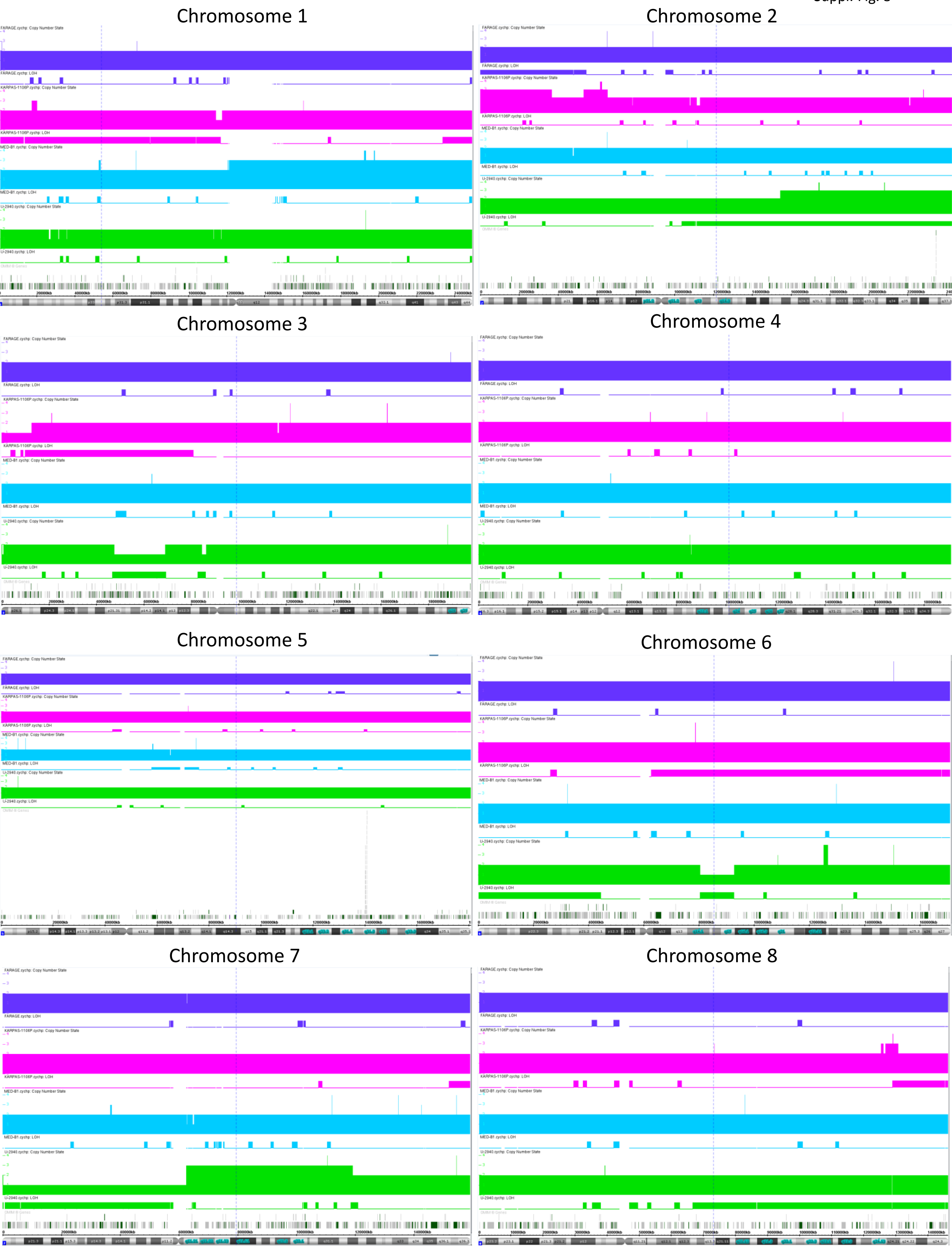
Chromosome 1
Chromosome 2
Chromosome 3
Chromosome 4
Chromosome 5
Chromosome 6
Chromosome 7
Chromosome 8
FARAGE.cychp: Copy Number State
FARAGE.cychp: LOH
KARPAS-1106P.cychp: Copy Number State
KARPAS-1106P.cychp: LOH
MED-B1.cychp: Copy Number State
MED-B1.cychp: LOH
U-2940.cychp: Copy Number State
U-2940.cychp: LOH

Chromosome 9

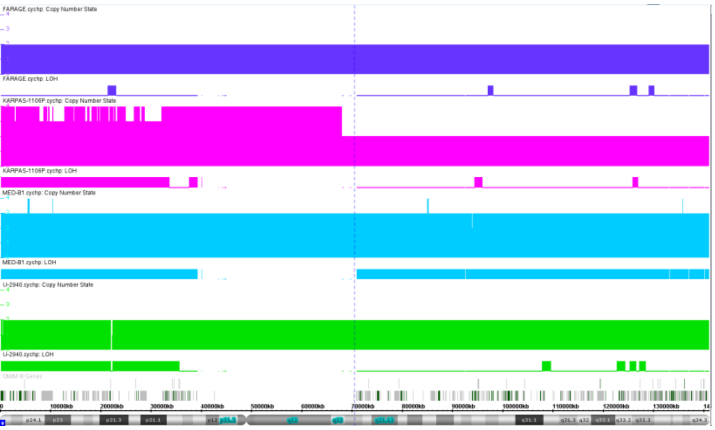

Chromosome 10

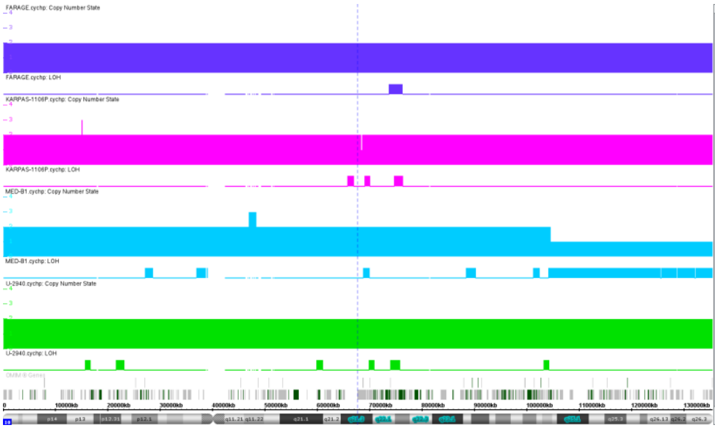

Chromosome 11

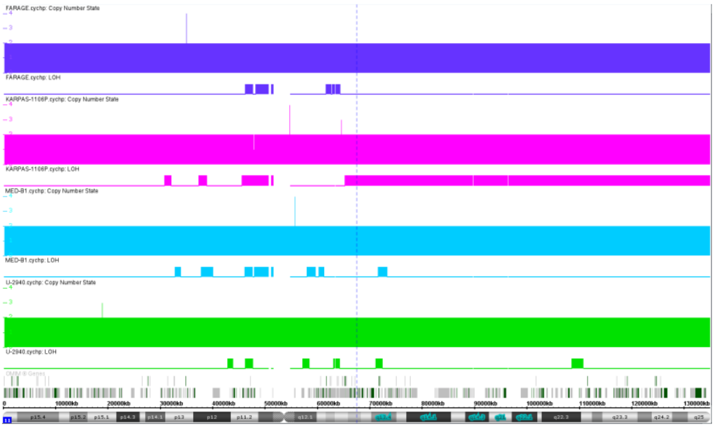

Chromosome 12

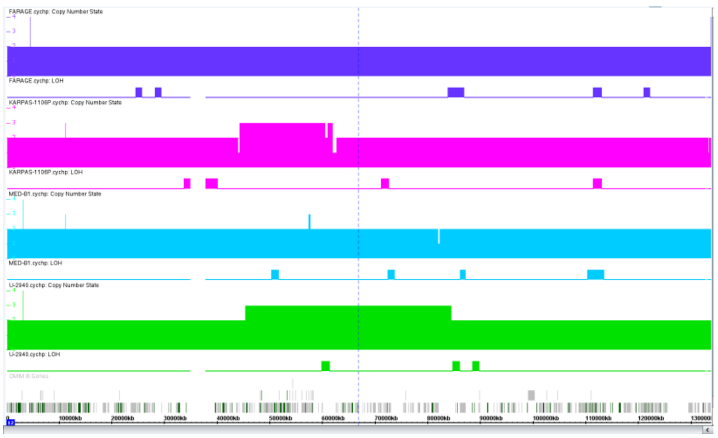

Chromosome 13

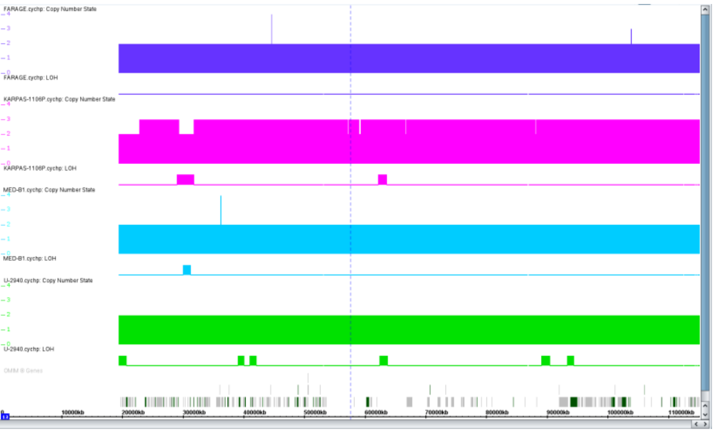

Chromosome 14

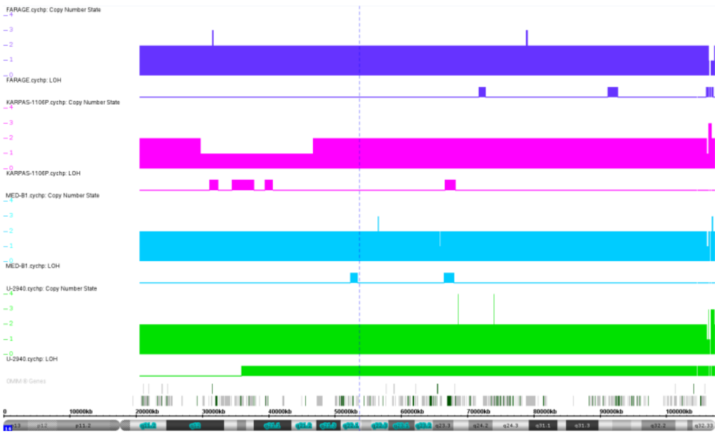

Chromosome 15

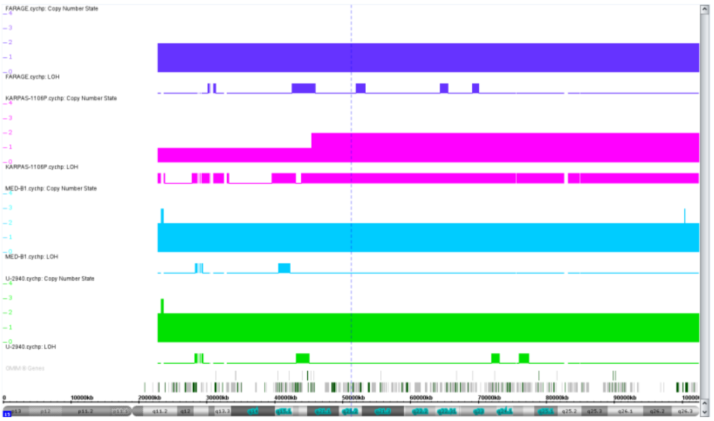

Chromosome 16

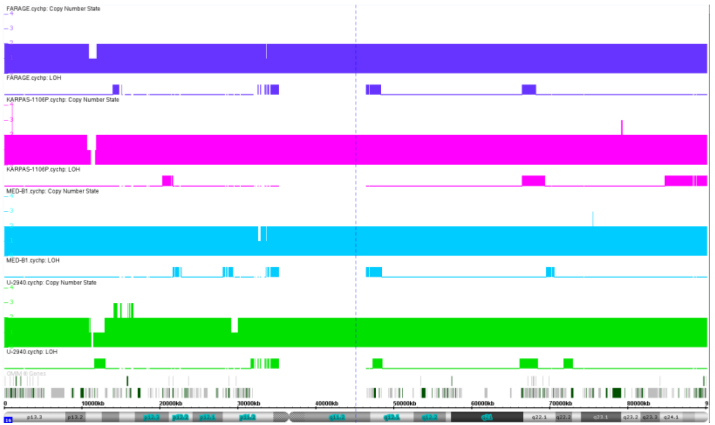

Suppl. Fig. 3 ctd

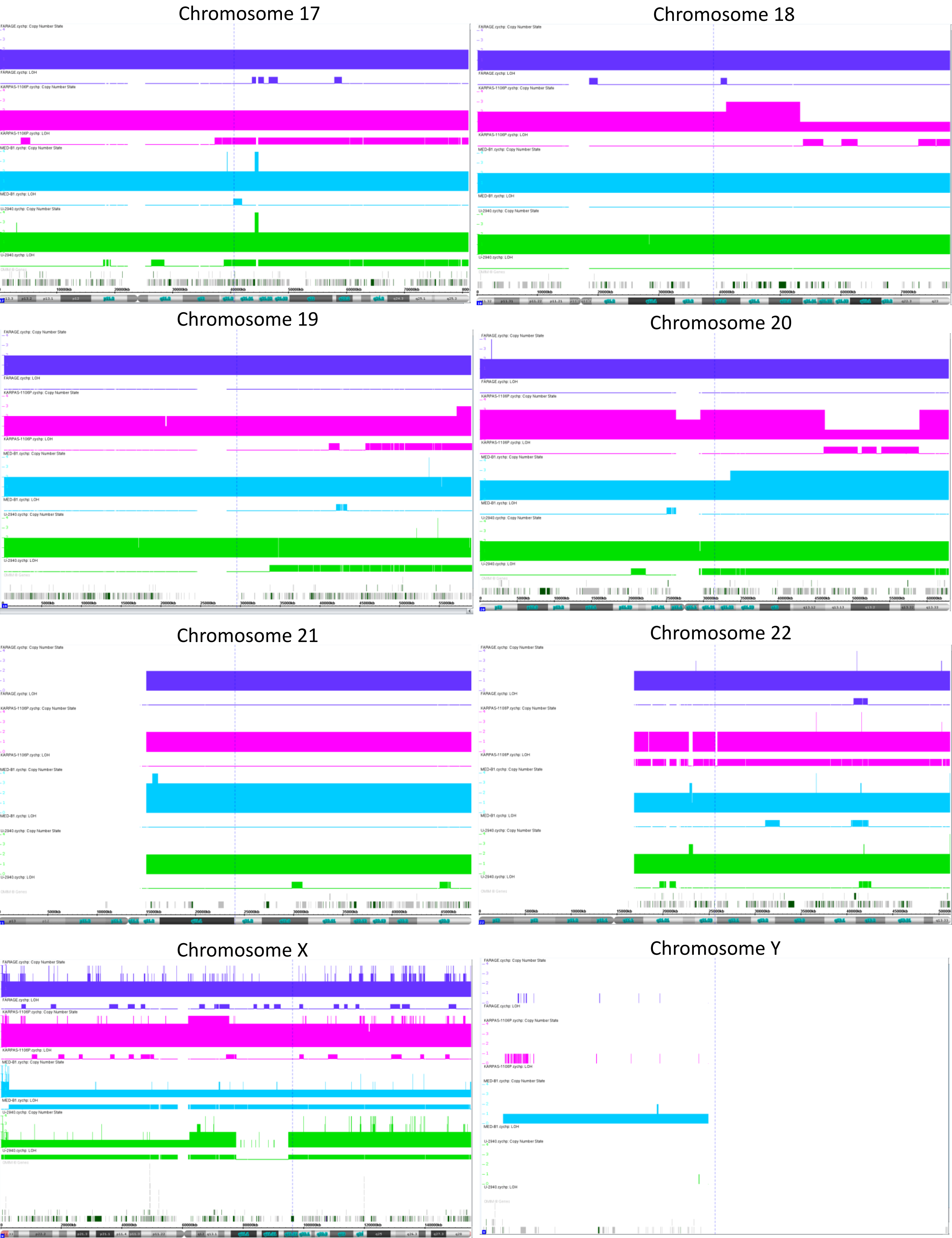

Supplement: S3 Fig — See legend to Fig 3. (PDF) [file pone.0139663.s003.pdf]
