# Supplementary material for: Genomic Landscape of Primary Mediastinal B-Cell Lymphoma Cell Lines
Source: PLoS One. 2015 Nov 23;10(11):e0139663. doi: 10.1371/journal.pone.0139663 (PMC4657880; doi:10.1371/journal.pone.0139663)
Supplement: S4 Fig — Shows global microarray expression for select genes in PMBL patients compared to cHL. Data extracted from ref [27]. (PDF) [file pone.0139663.s004.pdf]

Suppl Fig. 4A

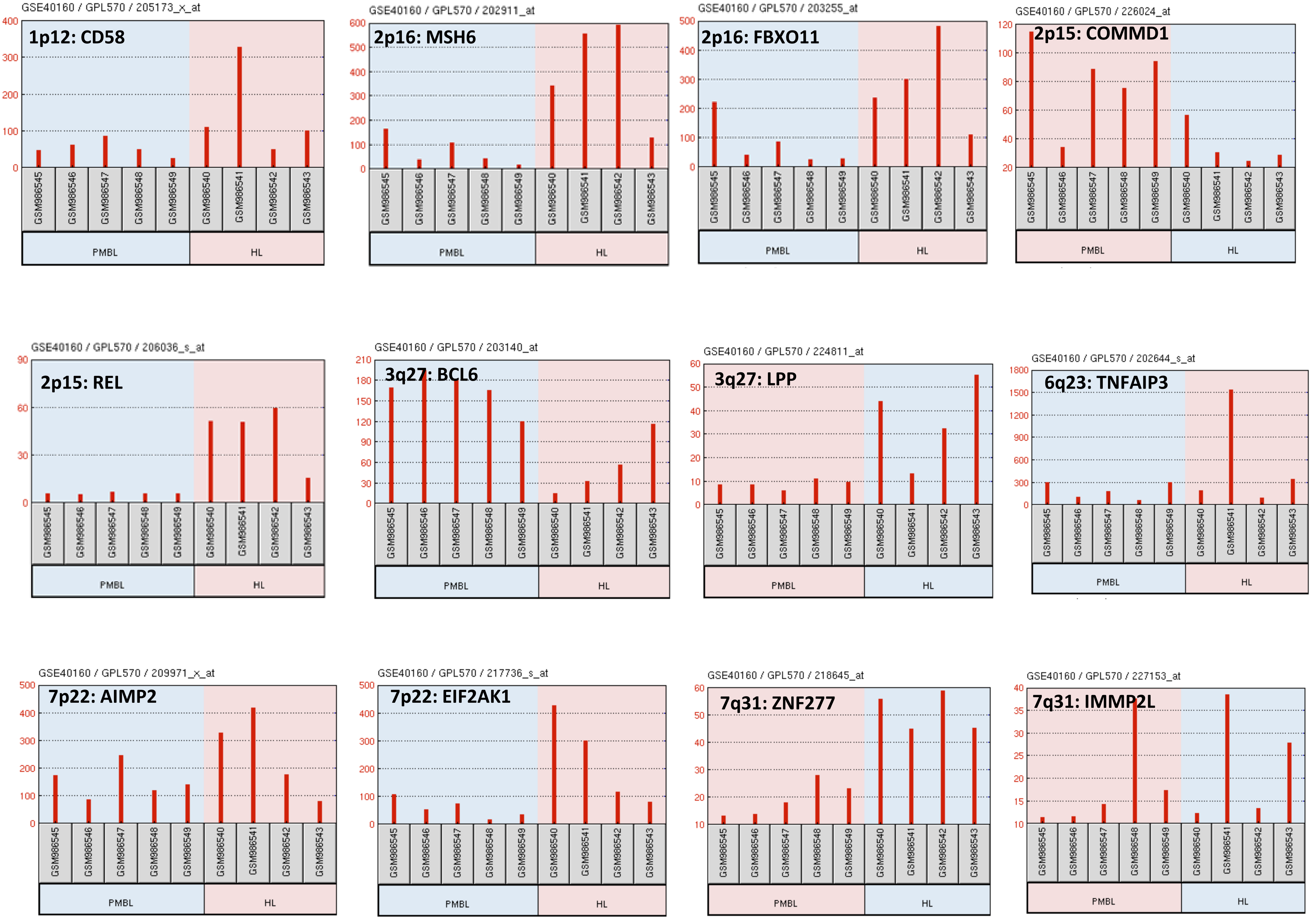
GSE40160 / GPL570 / 205173_x_at
1p12: CD58
GSE40160 / GPL570 / 202911_at
2p16: MSH6
GSE40160 / GPL570 / 203255_at
2p16: FBXO11
GSE40160 / GPL570 / 226024_at
2p15: COMMD1
GSE40160 / GPL570 / 206036_s_at
2p15: REL
GSE40160 / GPL570 / 203140_at
3q27: BCL6
GSE40160 / GPL570 / 224811_at
3q27: LPP
GSE40160 / GPL570 / 202644_s_at
6q23: TNFAIP3
GSE40160 / GPL570 / 209971_x_at
7p22: AIMP2
GSE40160 / GPL570 / 217736_s_at
7p22: EIF2AK1
GSE40160 / GPL570 / 218645_at
7q31: ZNF277
GSE40160 / GPL570 / 227153_at
7q31: IMMP2L
GSM986545
GSM986546
GSM986547
GSM986548
GSM986549
GSM986540
GSM986541
GSM986542
GSM986543
PMBL
HL

Suppl Fig. 4B

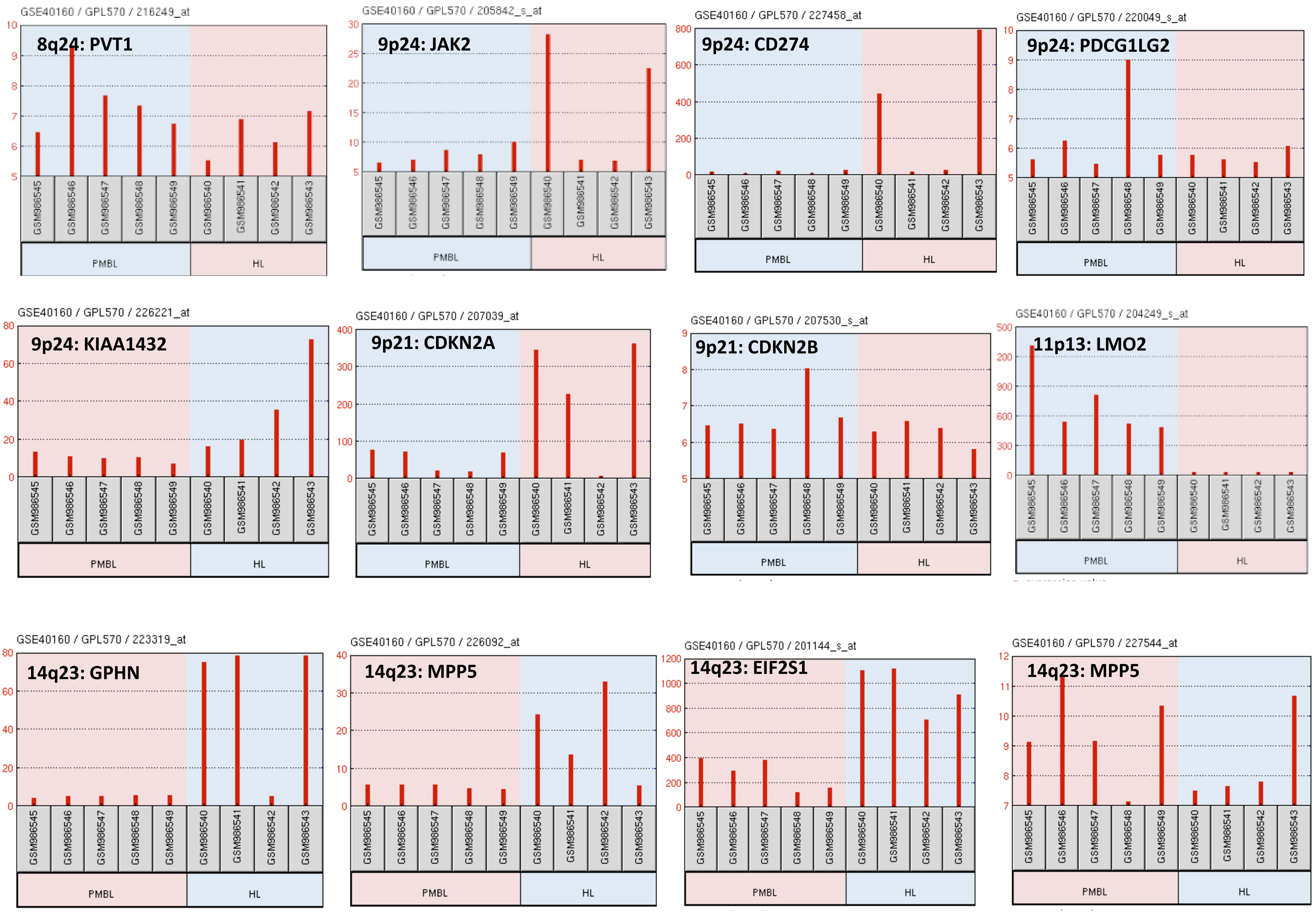
GSE40160 / GPL570 / 216249_at
8q24: PVT1
GSE40160 / GPL570 / 205842_s_at
9p24: JAK2
GSE40160 / GPL570 / 227458_at
9p24: CD274
GSE40160 / GPL570 / 220049_s_at
9p24: PDCG1LG2
GSE40160 / GPL570 / 226221_at
9p24: KIAA1432
GSE40160 / GPL570 / 207039_at
9p21: CDKN2A
GSE40160 / GPL570 / 207530_s_at
9p21: CDKN2B
GSE40160 / GPL570 / 204249_s_at
11p13: LMO2
GSE40160 / GPL570 / 223319_at
14q23: GPHN
GSE40160 / GPL570 / 226092_at
14q23: MPP5
GSE40160 / GPL570 / 201144_s_at
14q23: EIF2S1
GSE40160 / GPL570 / 227544_at
14q23: MPP5
GSM986545
GSM986546
GSM986547
GSM986548
GSM986549
GSM986540
GSM986541
GSM986542
GSM986543
PMBL
HL

Suppl Fig. 4C

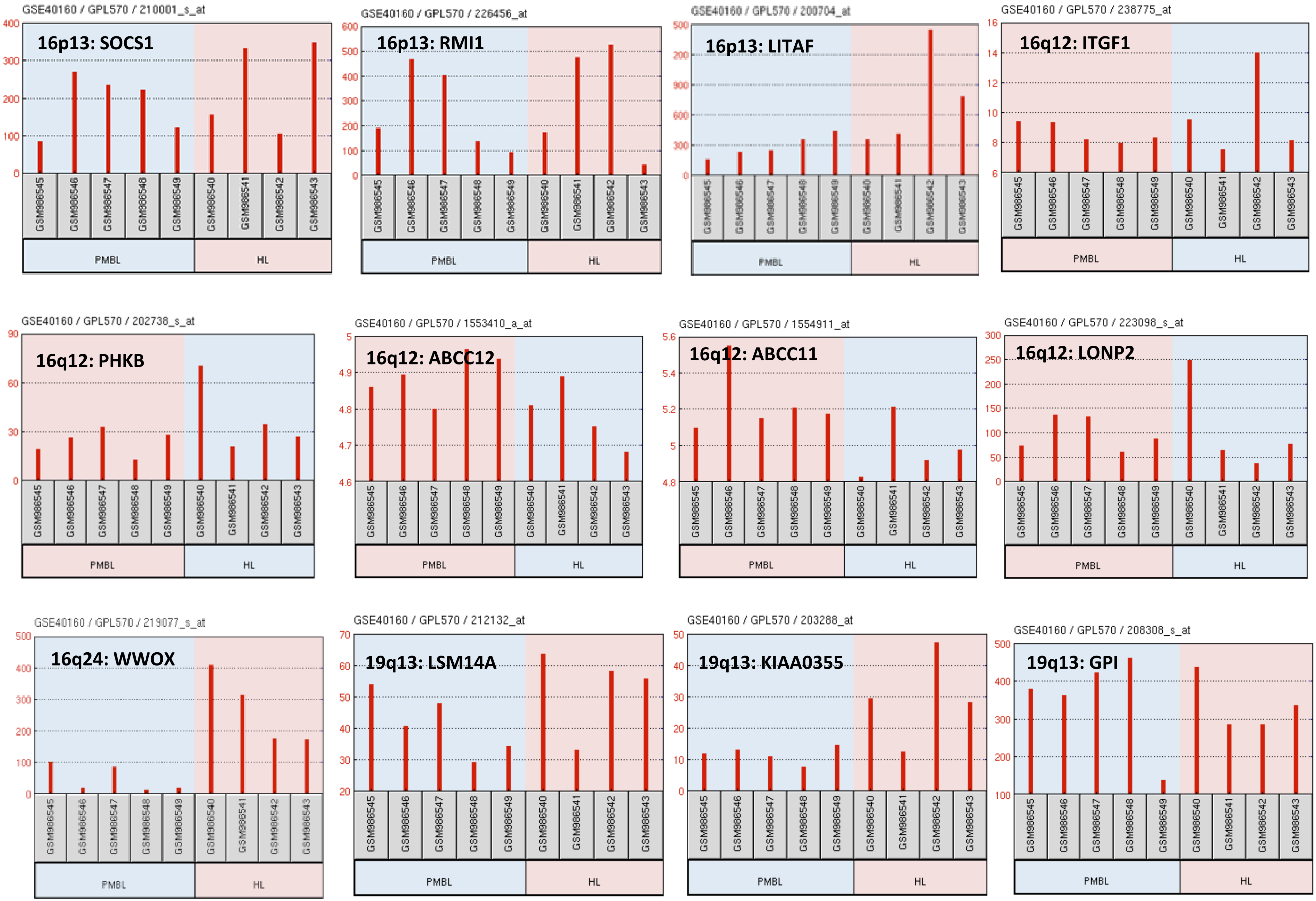
GSE40160 / GPL570 / 210001_s_at
16p13: SOCS1
GSE40160 / GPL570 / 226456_at
16p13: RMI1
GSE40160 / GPL570 / 200704_at
16p13: LITAF
GSE40160 / GPL570 / 238775_at
16q12: ITGF1
GSE40160 / GPL570 / 202738_s_at
16q12: PHKB
GSE40160 / GPL570 / 1553410_a_at
16q12: ABCC12
GSE40160 / GPL570 / 1554911_at
16q12: ABCC11
GSE40160 / GPL570 / 223098_s_at
16q12: LONP2
GSE40160 / GPL570 / 219077_s_at
16q24: WWOX
GSE40160 / GPL570 / 212132_at
19q13: LSM14A
GSE40160 / GPL570 / 203288_at
19q13: KIAA0355
GSE40160 / GPL570 / 208308_s_at
19q13: GPI
GSM986545
GSM986546
GSM986547
GSM986548
GSM986549
GSM986540
GSM986541
GSM986542
GSM986543
PMBL
HL
