# Supplementary material for: Genomic Landscape of Primary Mediastinal B-Cell Lymphoma Cell Lines
Source: PLoS One. 2015 Nov 23;10(11):e0139663. doi: 10.1371/journal.pone.0139663 (PMC4657880; doi:10.1371/journal.pone.0139663)
Supplement: S1 File — (DOCX) [file pone.0139663.s005.docx]

**S1 Table: STR Profiling Data**

|  | Polymorphic alleles | | | | | | | | | | | | | | | | | |
| --- | --- | --- | --- | --- | --- | --- | --- | --- | --- | --- | --- | --- | --- | --- | --- | --- | --- | --- |
| Cell Line | D5 | | D13 | | D7 | | D16 | | vWA | | TH01 | | TPOX | | CSF1 | | Amel | |
| FARAGE | 12 | 12 | 11 | 13 | 12 | 12 | 11 | 12 | 14 | 15 | 8 | 9 | 9 | 9 | 11 | 12 | X | X |
| KARPAS-1106P | 8 | 14 | 9 | 11 | 9 | 13 | 9 | 9 | 18 | 20 | 6 | 6 | 9 | 11 | 10 | 12 | X | X |
| MEDB-1 | 10 | 11 | 11 | 15 | 9 | 10 | 10 | 12 | 17 | 18 | 8 | 8 | 8 | 11 | 11 | 11 | X | Y |
| U-2940 | 11 | 12 | 8 | 10 | 10 | 12 | 10 | 12 | 17 | 17 | 7 | 9.3 | 8 | 12 | 12 | 12 | X | X |

**S2 Table: Primers for RqPCR**

**ACTR2:** actin-related protein 2 homolog

Forward primer: 5´-TTGCTGGGAGGGATATAACTAG-3´ ACTR2 fw

Reverse primer: 5´-AGCTTCTGGTGCTTCAAATCTC-3´.ACTR2 rv

Product length 242

**RAF1:** RAF proto-oncogene serine/threonine-protein kinase

Forward primer: 5´-AGATTGGAATACTGATGCTGCG-3´ RAF1 fw

Reverse primer: 5´-AATTTGTAGCCACAAGTCTGAC-3´ RAF1 rv

Product length 180

**ACTR2-RAF1** product length 287

---------------------------------------------------------------------------------------------------------

**CD274::** Homo sapiens CD274 molecule (CD274), transcript variant 2, mRNA

Forward primer 5´-CCTACTGGCATTTGCTGAACG-3´ CD274 fw

Reverse primer 5´-AGACAATTAGTGCAGCCAGG-3´ CD274 rv

Product length 135

**CDKN2A:** Homo sapiens cyclin-dependent kinase inhibitor 2A (CDKN2A), transcript variant 1, mRNA

Forward primer 5´-CACCGAATAGTTACGGTCGGA-3´ CDNK2A fw

Reverse primer 5´-CACGGGTCGGGTGAGAGTG-3´ CDNK2A rv

Product length 128

**CDNK2B:** Homo sapiens cyclin-dependent kinase inhibitor 2B (p15, inhibits CDK4) (CDKN2B), transcript variant 1, mRNA

Forward primer 5´-CTAGTGGAGAAGGTGCGACAG-3´ CDNK2B fw

Reverse primer 5´-CCATCATCATGACCTGGATCG-3´ CDNK2B rv

Product length 94

**CIITA:** Homo sapiens class II, major histocompatibility complex, transactivator (CIITA), transcript variant 2, mRNA

Forward primer 5´-ATTTGCCCCTCTGGATTGGG-3´ CIITA fw

Reverse primer 5´-TGCTGCCTGAAGTAGCTTGG-3´ CIITA rv

Product length 132

**COMMD1:** Homo sapiens copper metabolism (Murr1) domain containing 1 (COMMD1), mRNA

Forward primer 5´-CGGAGCCAGCTATATCCAGAG-3´ COMMD1 fw

Reverse primer 5´-AGCAGTCAAGAATGCCTCCAG-3´ COMMD1 rv

Product length 129

**JAK2:** Homo sapiens Janus kinase 2 (JAK2), mRNA

Forward primer 5´-GGGGTTTTCTGGTGCCTTTG-3´ JAK2 fw

Reverse primer 5´-GGGTCATACCGGCACATCTC-3´ JAK2 rv

Product length 120

**LITAF:** Homo sapiens lipopolysaccharide-induced TNF factor (LITAF), transcript variant 2, mRNA

Forward primer 5´-AGGGCATGAATCCTCCTTCG-3 LITAF fw

Reverse primer 5´-CCAAAAAGGTGATGGGGTGC-3´ LITAF rv

Product length 105

**REL:** Homo sapiens v-rel avian reticuloendotheliosis viral oncogene homolog (REL), mRNA

Forward primer 5´-GCACGTTCATGCTTTGTTTCCA-3´ C-REL fw

Reverse primer 5´-TCATTCAGCTGTTTTTCAGGGA-3´ C-REL.rv

Product length 130

**SOCS1:** Homo sapiens suppressor of cytokine signaling 1 (SOCS1), mRNA

Forward Primer: 5´-AGACCCCTTCTCACCTCTTG-3´ SOCS1 fw

Reverse Primer: 5´-AGAGGTAGGAGGTGCGAGTT-3´ SOCSS1 rv

Product length 130
